# Psychosocial dynamics of suicidality and nonsuicidal self-injury: a digital linguistic perspective

**DOI:** 10.1038/s44184-025-00142-w

**Published:** 2025-07-08

**Authors:** Charlotte Entwistle, Katie Hoemann, Sophie J. Nightingale, Ryan L. Boyd

**Affiliations:** 1 Lancaster University, Lancaster, UK; 2 University of Liverpool, Liverpool, UK; 3 University of Kansas, Lawrence, KS USA; 4 KU Leuven, Leuven, Belgium; 5 University of Texas at Dallas, Dallas, TX USA

**Keywords:** Psychology, Human behaviour

## Abstract

Self-harm—encompassing suicidality and nonsuicidal self-injury (NSSI)—presents a critical public health concern, particularly as it is a major risk factor of death by suicide. Understanding the psychosocial dynamics of self-harm is imperative. Accordingly, in a large-scale, naturalistic study, we leveraged modern language analysis methods to provide a comprehensive perspective on suicidality and NSSI, specifically in the context of borderline personality disorder (BPD), where self-harm is particularly prevalent. We utilised natural language processing techniques to analyse Reddit data (i.e., BPD forum posts) of 992 users with self-identified BPD (combined *N* posts = 66,786). The present findings generated further insight into the psychosocial dynamics of suicidality and NSSI, while also uncovering meaningful interactions between the online BPD community and these behaviours. By integrating advanced computational methods with psychological theory, our findings provide a nuanced understanding of self-harm, with implications for clinical practice, clinical and personality theory, and computational social science.

## Introduction

Suicidality and nonsuicidal self-injury (NSSI)—comprising the broader construct of “self-harm”—represent some of the largest public health problems in today’s world^1^. Concerningly, self-harm is the most important risk factor for death by suicide^2–4^. Better understanding of self-harm is not only a moral imperative, but an essential societal goal, necessitating more comprehensive research and understanding to mitigate the profound repercussions. Individuals who engage in self-harm, and those experiencing mental health problems more broadly, are increasingly turning to online platforms to seek support^5^. The disclosure of self-harm on online platforms provides researchers new opportunities to study self-harm at large scale and, importantly, in naturalistic contexts. In particular, the analysis of natural language provides a powerful approach to understanding self-harm, given that one’s language use can provide insight into their underlying psychology, values, motivations, and behaviours^6,7^.

In the present study, we leverage modern natural language processing (NLP) methods to analyse large online mental health forums to investigate the psychosocial dynamics—that is, the internal, psychological and the external, relational processes that constitute our everyday lives—surrounding suicidality and NSSI, while also examining community dynamics in relation to these behaviours. We investigate suicidality and NSSI in the context of borderline personality disorder (BPD), focusing on online BPD communities. The value of studying self-harm in the context of BPD is apparent from the strong relationship between BPD and self-harm, with self-harm even conceptualised as a key marker for the detection of BPD^8^. Indeed, prevalence rates of NSSI have been reported to be around 90% among adults with BPD^9^. Likewise, suicidality commonly accompanies BPD, and in a chronic fashion^10,11^. Moreover, BPD is associated with high rates of suicide, reaching as high as 10%^10^. Online BPD communities therefore present a meaningful sample for studying self-harm, and can simultaneously help to unravel why this is so common and problematic in people with BPD, or severe dysfunction more generally.

To conceptualise our work, NSSI is defined broadly as an intentional act of causing oneself physical harm without suicidal intent; this can include direct NSSI behaviours (i.e., deliberate acts that cause physical damage to body tissue), such as cutting, burning, and hitting oneself, as well as indirect NSSI (i.e., behaviors that damage the body but do not involve the direct and deliberate destruction of body tissue), such as substance abuse and otherwise risky behaviours (e.g., dangerous driving; unsafe sex). By contrast, suicidality reflects a spectrum of suicide-relevant psychosocial processes^12^—both active and passive in nature—including cognitive processes like suicidal ideation (i.e., thoughts about suicide), affective dynamics related to suicide (e.g., feelings of not wanting to be alive), and suicidal behaviours, such as preparing and attempting suicide. Due to the far-reaching, naturalistic, person-centred approach to our work, and given that our goal is to better understand self-harm in the broadest sense, the adoption of flexible self-harm terminology—for example, “direct” and “indirect” NSSI as well as “active” and “passive” suicidality—is warranted. The use of flexible and inclusive definitions of self-harm permits the individuals covered by our study to define self-harm, and their experiences with it, in their own words, allowing us to investigate these phenomena in a more ecologically valid, individualised way. Vitally, our use of flexible, person-centred definitions of self-harm ensures that our analyses do not place artificial boundaries on what qualifies as self-harm.

As for what we already know about the psychosocial dynamics of self-harm, suicidality and NSSI share overlapping functions, including affect regulation^13,14^ and social functions^15,16^. Commensurate with this notion, traditional research has identified overlap in the psychosocial risk factors for each, in the context of BPD and more generally. For instance, a recent literature review uncovered psychological distress, affective instability/dysregulation, impulsivity, and social dysfunction as risk factors for both suicidality and NSSI^17^. Studies that have focused specifically on people with BPD have additionally found emotion-related impulsivity and dysfunctional cognitive processes (e.g., negative rumination) to be major risk factors of both suicidality and NSSI^18,19^. Comparing risk factors of suicidality and NSSI, an “all-or-nothing” (i.e., absolutist or dichotomous) thinking style has been evidenced to be a key risk factor for suicidality, but not for NSSI^20^. Moreover, affective dysregulation is a strong predictor of both suicidality and NSSI, but differs in the form that it takes; affective instability and heightened negative affect predict both suicidality and NSSI^17^, whereas dissociation (i.e., feelings of detachment from oneself, one’s surroundings, or one’s thoughts and feelings) is more reliably associated with NSSI^21^.

Existent research provides useful insight into risk factors of suicidality and NSSI, yet is limited by a heavy reliance on self-report measures and otherwise non-naturalistic methods. These forms of measurement are problematic for numerous reasons, such as memory distortion issues and social desirability biases^22,23^. More generally, individuals participating in such studies may simply lack the necessary insight into their own internal processes to accurately report on psychosocial correlates of suicidality and NSSI, which is even more likely in those who suffer identity disturbances^24^.

Overcoming constraints of self-report methods, there has been recent growth in research leveraging naturalistic language analysis techniques to study suicidality and NSSI. Two broad types of research have been carried out in this domain: research focused on predictive accuracy (i.e., NLP and machine learning tasks that aim to correctly identify the content of a message) and research directed towards psychological explanation (i.e., generating interpretable, psychologically meaningful results that generate greater understanding). Most of the work to date that has applied NLP methods to the study of self-harm has focused on predictive accuracy, including identifying linguistic markers of self-harm.

With respect to such predictive accuracy focused NLP work, linguistic features revealed to predict suicide and NSSI posts (i.e., at the document/post level) largely reflect the theory-based psychosocial risk factors of suicidality and NSSI, such as internalisation, social disconnection, and heightened negative emotion. That is, suicidality and NSSI posts are marked by high rates of self-focused language^25,26^—a widely established indicator of mental distress and depression^27^—and linguistic indicators of affective dysregulation (i.e., greater negative affect and fewer positive affect words^25,26,28,29^). Likewise, suicide-focused posts have been revealed to indicate social dysfunction (i.e., fewer we-words, you-words, and social words^25,29^) and cognitive biases (i.e., greater absolutist language, such as “always”, “totally”, reflecting all-or-nothing thinking^30^).

Extending beyond stable risk factors, researchers have also worked to determine *when* individuals are more likely to engage in self-harm. Considerable attention has been directed towards the affective dynamics surrounding self-harm—that is, fluctuations in mood and emotion and their relationship to self-harm. There is strong empirical evidence highlighting the role of affective dysregulation as a temporal precursor to suicidality and NSSI, in BPD and more generally, demonstrated in both more traditional research^8,31–33^ and NLP work^34–36^. Generally speaking, research has found heightened negative affect to precede both suicidality and NSSI, which typically decreases after the occurrence of these events^37^. These affective dynamics have largely been mirrored in NLP research. Specifically, studies typically show negative emotive language increases, and positive emotive language decreases, in weeks leading up to suicide-relevant events^35,36^. Notably, this pattern of affective dynamics is most consistent for suicidality, as it has been repeatedly evidenced in BPD and non-BPD contexts, albeit with stronger effects among individuals with BPD^38^.

In comparison, the affective dynamics surrounding NSSI are less reliably evidenced. Notably, a Four-function Model of NSSI has been proposed^31^, which positions NSSI as serving four types of affective (intrapersonal) and social (interpersonal) functions, of which are either negatively or positively reinforcing. Focusing on the intrapersonal/affective function, this model theorises that individuals engage in NSSI to reduce and relieve negative states and affect (negative reinforcement), and also to enhance positive internal states/affect (positive reinforcement). Some support for this model has been evidenced over recent years (see^32,33^ for recent reviews). Most of this support comes from evidence that heightened negative affect predicts subsequent engagement in NSSI, with some studies finding negative affect to then decrease following NSSI (as with suicidality)^32,33^. However, in contrast, other studies have reported negative affect to increase both prior to *and* following NSSI^32,33,39,40^. Moreover, some research has found intense feelings of dissociation to precede NSSI, which typically eases following NSSI^21^; a pattern that appears more prevalent among individuals with BPD^32^. As for positive affective dynamics surrounding NSSI, findings are highly mixed and inconclusive^32,33^.

Related research has expanded the investigation of precursors to self-harm to include other, non-affective psychosocial processes. Namely, interpersonal problems—typically in the form of isolation, rejection, or interpersonal conflict—has been established as a precursor to both suicidality^41^ and NSSI^32,42^ in individuals with BPD, relating to self-harm functions of both interpersonal negative and interpersonal positive reinforcement^31,32^. Consistent with this, research using NLP methods revealed indicators of social dysfunction (i.e., decreased socially orientated and connected language) to precede suicidality^34^. However, evidence for this trend is inconsistent^35,43^. Behavioural dysregulation, particularly impulsivity, has been identified as another key precursor to suicidality and NSSI^44^, which is mirrored in NLP work on suicidality^34^. Lastly, absolutist language^34^ and indicators of dysfunctional self-processes (e.g., heightened self-focused language^34,43^) have also been uncovered as temporal precedents to suicidality.

Building on this, in going a step beyond the majority of past work leveraging online behaviour to study self-harm, it is also important to consider another, relatively understudied critical dimension; the dynamics of the online communities being studied themselves (i.e., the context of the data^45^). Research investigating effects of online support communities—like the BPD support communities in the present study—is in its infancy, despite this becoming a rapidly popular method of seeking mental health support^5^.

Online support communities are often studied in a general sense, but typically not in the context of discrete clinical events, such as disclosures of self-harm. One study examined features of mental-health-support-seeking posts that generated more supportive community responses^46^. Posts (i.e., mental health disclosures) that were more positive, more sociable, more self-focused, and shorter received greater community support (in the form of more “upvotes”), whereas posts that were more negative emotive and contained more swear words received less community support (in the form of less “upvotes”)^46^. In the context of self-harm specifically, De Choudhury and colleagues^34^ examined interactions with online mental health communities as predictors of future use of a suicide support forum. Features of community interactions revealed to predict future suicide forum use included lower levels of engagement with the community and less support received from the community (i.e., less replies and upvotes in response to their posts).

The existing research highlights the importance of acknowledging and understanding dynamics of online communities when studying online behaviour, particularly in the context of mental health. However, significant gaps remain in how these interactions unfold within disorder-specific communities, such as BPD support communities. Moreover, while prior studies have examined the nature and/or effects of support-seeking posts, supportive responses, and community engagement, there is a lack of research exploring these dynamics in depth, particularly in relation to suicidality and NSSI.

Taken in all, the research discussed has uncovered major gaps in the literature with respect to understanding suicidality and NSSI via naturalistic computational methods. To summarise the most pivotal gaps, first, research using NLP to detect or predict suicidality or NSSI from online data has been criticised due to its “black box” nature, with studies often lacking in construct validity and integration of psychological theory/perspective, thus restricting meaningful psychological insight^47^. Second, none of the NLP studies discussed were conducted in the context of BPD. Given the new knowledge NLP methods have provided in relation to self-harm in general, it would be propitious to take the best aspects of this research and bring them to bear on BPD, where self-harm is problematically prevalent. It can be seen from existing research that psychosocial dynamics of self-harm may differ in individuals with BPD, or those suffering severe dysfunction more broadly; thus, examining self-harm in people with BPD using sophisticated NLP methods could yield further insight into why self-harm is so prevalent in those suffering severe personality dysfunction. Third, nearly all of the NLP studies discussed (bar one^43^) have not examined the after-effects of self-harm, which is critical for painting a fuller psychological picture. Fourth, the language-based research discussed has exclusively focused on suicidality, with no psychologically insightful research applying NLP methods to NSSI. Finally, research into the dynamics of online mental health support communities in relation to mental health outcomes is scarce, and we are not aware of any research that has examined interactions between online BPD communities and suicidality or NSSI.

In the present work, we address the above research gaps by leveraging naturalistic language analysis methods to analyse large online BPD forums, investigating the psychosocial dynamics of suicidality and NSSI in a critical population. Acknowledging the context of the data^45^, we also examine online BPD community dynamics in relation to suicidality and NSSI. Specifically, we analyse data extracted from the social media platform Reddit, leveraging BPD discussion/support forums (i.e., “subreddits”). By integrating computational linguistic methodological approaches with those focused on psychological explanation, we develop a “middle-ground” relative to past work, incorporating psychological theory and generating meaningful psychological insight. We aim to address three central research questions:

RQ1: In what ways are the psychosocial dynamics of suicidality and NSSI evident in verbal behaviour?

RQ2: What features characterise the online BPD community’s interaction with disclosures of suicidality and NSSI?

RQ3: How might the online BPD community interact with the psychosocial dynamics preceding suicidality and NSSI events to shape the outcome of these events?

We address the central research question—RQ1—by examining associations between key linguistic features and frequencies of disclosures of suicidality and NSSI, shedding light onto the person-level psychosocial correlates of suicidality and NSSI. Further, we address RQ1 in greater depth by investigating changes in key linguistic features (i.e., linguistic trajectories) across the weeks preceding and following suicidality and NSSI events, permitting insight into the *temporal* psychosocial dynamics in proximity to self-harm. We address RQ2 and RQ3 through examining interactions between the online BPD community and suicidality and NSSI disclosure posts (RQ2), and with the key linguistic features found to temporally precede suicidality and NSSI (RQ3).

## Methods

This study was approved by the Faculty of Science and Technology Research Ethics Committee (FSTREC) at Lancaster University.

### Data Overview

Data were collected from the widely used, publicly available social media platform Reddit. Briefly described, Reddit is a discussion forum composed of “subreddits” dedicated to specific topics (e.g., sports teams, food, etc.). Within Reddit, users make posts (“submissions”) relevant to the given topic, responding through chains of “comments”.

As part of a broader investigation of behaviour in BPD—creating the BPD and Behaviour Reddit Dataset (BBRD^48^)—data were extracted from the *r/BPD* and *r/BorderlinePDisorder* subreddits, whereby people commonly identify as having BPD and seek support from, and build connections with, others with BPD. These subreddits were selected as they are the dominant forums for people with BPD to discuss their disorder on Reddit, with the two subreddits combined comprising over 300,000 individual community members to date, making them among the largest, if not the largest, online BPD communities. All posts (including submissions and comments), along with the associated meta-data (e.g., number of replies to posts, post scores) made between October 2011 (when the subreddits were first formed) and August 2019 (when data were extracted from Reddit) were extracted from the *r/BPD* and *r/BorderlinePDisorder* subreddits from a larger Reddit database^49^. In total, 607,559 posts from 52,369 individual users were extracted.

### Data Refinement and Extraction

The examination of linguistic (and psychosocial) trajectories over time necessitates that users have made multiple posts within the BPD subreddits, as just one or two posts would not permit the analysis of change over time. Consequently, only users who had made a minimum of 10 posts within the BPD subreddits (over the full dataset timespan) were retained, which left 453,761 posts from 18,276 individual users. Given the large dataset, setting a minimum criterion of 10 posts ensures an inclusive sample with a large number of users while also increasing the likelihood of retaining sufficient data—considering further pre-processing requirements—for meaningful assessment of individuals’ temporal psychosocial dynamics.

We further refined our dataset by selecting posts by only individuals who self-identified as having BPD. To do this, posts made to the BPD subreddits were manually inspected and coded by trained raters to identify those who self-identified as having BPD, with statements like “my BPD diagnosis” and “diagnosed with BPD”. This manual coding was aided by an automated, custom-made “BPD diagnosis” dictionary to highlight posts containing such phrases—a commonly adopted approach to identifying users with mental health conditions on online platforms^43^. Two separate raters coded each text, and any disagreements were clarified by a third researcher (the first author). Only texts where users unambiguously self-identified as having BPD were classified as the individual having the disorder. We aimed to extract a total of 1000 users with self-identified BPD, so as to generate a large sample that was also feasible with the time constraints that accompany manual coding. Although we initially achieved this sample size goal, further manual quality checks indicated that some of the 1000 users exhibited less clear BPD identification. Consequently, the coding process resulted in 992 users identified as having BPD, with a combined total of 97,787 posts made across them. This BPD classification coding had good inter-rater reliability (α = 0.74).

As Reddit is largely an anonymous platform, we did not have access to users’ demographic information to characterise the sample. Accordingly, posts made by the 992 users identified as having BPD were manually coded to extract demographic characteristics (e.g., age, gender). The demographic coding process was carried out in the same way as the BPD classification coding process.

Among the goals of the larger investigation was to identify key (time-stamped) behaviours in users’ posts relevant to the construct of BPD, permitting detailed behavioural analytics. Subsequently, a coding framework guided the coding of nine BPD/psychopathology-relevant behaviours/events in users’ posts, including self-harm behaviours, medication- and therapy-related behaviour, substance use, impulsive behaviour, social interactions, and emotion-relevant behaviours/events. The coding of behaviours/events was consistent with the coding procedure described above; also aided by an automated dictionary stratification approach. That is, texts selected for manual coding were those which contained the most keywords related to a particular behaviour/event. For instance, examples of key words/phrases used for suicidality include “want to die” and “feel suicidal”. Resulting from this dictionary-based stratification process, of the 97,787 posts made by the 992 users with BPD, 9106 were selected for manual coding of behaviours and events. Of the behaviours/events coded, it is those related to self-harm (i.e., suicidality and NSSI) that are of interest for the present study.

In the coding process, suicidality was characterised by suicidal ideation/feelings (i.e., thoughts and feelings related to suicide) and suicidal behaviour (primarily suicide attempts), and separated into recent (i.e., within the same week for suicidal behaviour and on the day of writing for ideation) and past occurrences. NSSI was broken down into engagement in NSSI (including both direct and indirect forms) and urge for NSSI, and again into recent (within the same week) and past occurrences. We adopted broad inclusion criteria in coding the suicidality and NSSI categories, in accordance with the broad definitions outlined in the Introduction. For further detail, the coding guidance document provided to guide (and supplement comprehensive training) the coding of self-harm events is presented in Supplemental Materials A. There was generally high inter-rater agreement for the coding of demographics and behaviours/events (see Supplemental Materials B, Table S1).

### Language Pre-Processing and Analysis

Language data were cleaned and prepared for analysis according to standard guidelines^50^—formatting errors, common misspellings, and elongations were corrected, URLs were simplified, quotes were removed, and all texts that contained <25 words were removed from the dataset to ensure validity of measurement, along with all duplicate texts. Following this cleaning process, 66,786 individual posts remained (from 992 unique users).

To address our research questions (particularly RQ1 and RQ3), language-based psychological features were extracted using the latest version of the Linguistic Inquiry and Word Count (LIWC-22) software^51^. Briefly described, LIWC relies on an internal dictionary that maps words and phrases to psychologically meaningful categories, with the output produced by LIWC consisting of relative frequencies (i.e., percentages) of each category within each text. Despite its simplicity, LIWC has been extensively well-validated as to its utility in modelling mental health relevant language^52^.

For the purposes of the present study, we were primarily interested in the linguistic features that are closely related to self-harm, as well as the construct of BPD and psychopathology more broadly. Accordingly, the selection of linguistic features for inclusion was based on previous research findings that have evidenced their predictive validity in relation to suicidality, NSSI, or psychopathology in general, most of which have been described in the introduction of this article. A total of 16 linguistic features (derived from LIWC) were selected for inclusion, which we mapped onto four broad psychosocial dimensions: self-processes, emotion processes, social processes, and cognitive processes (reflecting core areas of dysfunction in BPD and psychopathology more broadly^53^). A list of the selected LIWC variables and their mapping onto each of the four dimensions, along with references to justify their inclusion, is presented in Table 1.

**Table 1:** Included LIWC Variables Mapped on to Psychosocial Dimensions Related to Psychopathology, with Supporting Empirical Evidence

Psychosocial Dimension	LIWC Category	Example Words	Direction of Association	Example Reference(s)
Self-processes/ functioning	I-words	I, me, my	-	Sierra et al.^25^ Uban et al.^26^ Tackman et al.^27^
	Negations	no, not, didn’t	-	Ramírez-Cifuentes et al.^28^ Coppersmith et al.^65^
Emotion processes/ functioning	Positive emotion	happy, excited, love	+	Glenn et al.^35^ Sierra et al.^25^
	Negative emotion	sad, hate, hurt	-	De Choudhury et al.^34^ Sierra et al.^25^ Uban et al.^26^
	Anxiety	anxious, fear, worry	-	De Choudhury et al.^34^ Lyons et al.^52^ Ramírez-Cifuentes et al.^28^
	Sadness	depressed, cry, upset	-	Glenn et al.^35^ Lyons et al.^52^
	Anger	Angry, mad, frustrated	-	Coppersmith et al.^43^ Glenn et al.^35^ Uban et al.^26^
	Swear words	shit, fuck, damn	-	Coppersmith et al.^65^
Social processes/ functioning	We-words	we, our, us	+	Coppersmith et al.^65^ Lyons et al.^52^ Sierra et al.^25^
	You-words	you, your, yourself	+	De Choudhury et al.^34^ Sierra et al.^25^
	Shehe-words	he, she, her	Mixed	De Choudhury et al.^34^ Lyons et al.^52^
	They-words	they, their, them	Mixed	De Choudhury et al.^34^ Lyons et al.^52^
	Affiliation	together, social, collectively	+	Ramírez-Cifuentes et al.^28^
	Social references	you, we, her	+	Aldhyani et al.^29^ Sierra et al.^25^
Cognitive processes/ functioning	Cognitive processes	think, puzzle, solve	Mixed	Aldhyani et al.^29^ Ramírez-Cifuentes et al.^28^
	Absolutism/all-none	always, never, definitely	-	Al-Mosaiwi & Johnstone^30^ De Choudhury et al.^34^

*Note*. This table shows the LIWC variables selected for inclusion in the present study and their mapping on to four broad psychosocial dimensions related to psychopathology. Example words from the LIWC22 dictionary are presented for each of the linguistic categories. The direction of association column shows the direction (i.e., positive [+] versus negative [-]) of the associations between the LIWC variables and the broader psychosocial dimensions based on previous research (presented in the “Example References” column), generally in the context of self-harm.

## Results

### Descriptive Analysis of Sample

We first characterise the posting behaviour of the 992 users with self-identified BPD; the descriptive results reported here reflect the sample characteristics following data pre-processing (i.e., after removing posts < 25 words and duplicate posts). Following the removal of posts < 25 words and duplicate posts, the total number of posts (including submissions and comments) of each of these users in the BPD subreddits ranged from 1–1958, with an average of 67.32 posts per user (*SD* = 106.58) and an average of 114.08 words per post (*SD* = 136.70). The overall duration of time from users’ first to their last post ranged from 0 to 2,481 days (6 years, 9 months, 17 days), with an average duration of 392.29 days (1 year, 27 days; *SD* = 407.07 days). Refer to Supplemental Materials C for descriptive illustrations of users’ posting behaviour.

The demographic coding procedure (described in the Methods section) allowed us to extract some demographic information to characterise the sample (see Table 2).

**Table 2:** Sociodemographic Characteristics of BPD Reddit Sample (*N* = 992 Users)

Characteristic	Mean	*SD*
Age (*n* = 260; 26.21%)	27.87	8.06

	*n*	%
Gender (*n* = 394; 39.71%)		
Female	237	60.15
Male	146	37.06
Non-binary	11	2.79
Country of residence (*n* = 294; 29.64%)		
UK	87	29.59
US	75	25.51
Canada	55	18.71
Australia	29	9.86
Other European country	34	11.56
Other non-European country	14	4.76
Religion (*n* = 102; 10.28%)		
Non-religious/atheist	48	47.06
Religious	39	38.24
Spiritual	7	6.86
Agnostic	8	7.84
Relationship status (*n* = 700; 70.56%)		
Single	156	22.29
In a relationship	378	54.00
Married	140	20.00
Divorced/separated	26	3.71

*Note*. The *n*s and percentages provided alongside each of the demographic categories reflect the total number of users (and percentage of the sample) we were able to extract each demographic variable for.

As for the behavioural coding, Table S2 in Supplemental Materials D shows the frequencies of suicidality and NSSI events manually coded, broken down into past and recent occurrences. Overall (including all subcategories), 1290 events (from 497 unique users) were captured for suicidality and 678 (from 319 unique users) for NSSI.

### RQ1: In what ways are the psychosocial dynamics of suicidality and NSSI evident in verbal behaviour? Trait-level linguistic features of self-harm

To examine the linguistic features of suicidality and NSSI, in a descriptive fashion, we conducted two-tailed, bivariate Spearman’s Rho correlation analyses between users’ total frequencies of disclosures of suicidality (including suicidal ideation/feelings/behaviours) and NSSI (including past and recent events) and mean scores (across all posts) for the 16 linguistic categories derived from LIWC. Spearman’s Rho correlations were chosen over Pearson’s due to the dataset comprising non-normally distributed frequency data. All posts coded for mention of suicidality or NSSI (*N* = 1968) were excluded from this analysis to ensure that the results are representative of users’ general language and do not simply reflect language patterns associated with suicidality/NSSI disclosures. All 992 users were included in this analysis. Note that although numerous analyses (i.e., multiple tests) are carried out in this project, we have not corrected for multiple comparisons given that this is descriptive work and involves individual testing only (i.e., testing individual relationships between individual variables). In this case, each test result only provides one opportunity to make a Type I error, and so alpha adjustment is not appropriate^54^.

Results from the correlation analyses are presented in Table 3. Linguistic indicators of dysfunctional self-processes (i.e., higher rates of first-person singular pronouns, or “I-words”) were positively associated with the frequency of both recent suicidality and NSSI. However, negations were only positively associated with recent suicidality.

**Table 3:** *Spearman’s Rho Correlations Between Mean Language Variable Scores and Suicidality and Nonsuicidal Self-Injury (NSSI) Frequencies (N* = *992 Users)*

LIWC Variable	Past Suicidality	Recent Suicidality	Past NSSI	Recent NSSI
I	0.02	0.16***	0.06^†^	0.11***
Negations	0.03	0.07*	0.04	0.02
Positive emotion	0.04	0.05	0.04	−0.04
Negative emotion	0.06^†^	0.14***	0.05	0.12***
Anxiety	0.07*	0.05	0.07*	0.05
Sadness	0.07*	0.12***	0.08*	0.09**
Anger	0.04	0.13***	0.04	0.09**
Swear	0.04	0.11***	0.03	0.03
We	0.02	−0.03	0.01	−0.02
You	−0.02	−0.06^†^	−0.02	−0.06^†^
Shehe	−0.02	−0.03	0.00	0.03
They	0.02	0.03	0.00	0.02
Affiliation	−0.01	−0.04	−0.04	0.04
Social references	−0.03	−0.06^†^	−0.06^†^	−0.06^†^
Cognitive processes	−0.09**	−0.01	−0.05	−0.10**
Absolutism	0.04	0.11***	0.00	0.01

***p < 0.001, **p < 0.01, *p < 0.05, ^†^*p* < 0.10.

*Note*. All tests are two-tailed. Language variable scores reflect users’ mean LIWC22 category scores from the BPD subreddits, excluding posts coded for suicidality or nonsuicidal self-injury (NSSI) of any nature (past or recent). Mean language scores were correlated with users’ overall frequency of suicidality/NSSI disclosures.

Markers of affective dysfunction were also associated with the frequency of disclosures of both suicidality and NSSI. Overall negative emotion, sadness, and anger words were positively associated with the frequency of recent suicidality and NSSI. In comparison, anxiety words were only positively associated with the frequency of past occurrences of these events. Swear words were positively correlated with the frequency of recent suicidality only. No associations emerged for positive emotion words.

No statistically significant associations were found for linguistic markers of social dysfunction. However, you-words (i.e., second-person pronouns) and social references were negatively associated with frequencies of recent suicidality and NSSI by trend.

As for cognitive functioning, negative associations emerged between cognitive processing language and frequencies of past suicidality and recent NSSI. Absolutist language was positively associated with the frequency of recent suicidality only.

Variations of these correlation analyses—whereby outliers were removed, and users’ total number of posts were controlled for—portray the same patterns as those reported here (see Supplemental Materials E).

### RQ1: In what ways are the psychosocial dynamics of suicidality and NSSI evident in verbal behaviour? Temporal linguistic trajectories surrounding self-harm

To better understand the temporal psychosocial dynamics surrounding suicidality and NSSI, we examined language changes in the weeks immediately preceding and following disclosures of recent suicidality and NSSI. To do this, we created a subset of the dataset, targeting posts that were coded for recent suicidality (including suicidal ideation/feelings/behaviours) and NSSI. As shown in Table S2 (Supplemental Materials D), this included 600 cases of recent suicidality (the large majority of which relate to suicidal thoughts and feelings, as opposed to suicidal behaviour; see Table S2 for a detailed breakdown) and 148 cases of recent NSSI. From there, we extracted mean linguistic feature scores of all posts 3 weeks preceding suicidality/NSSI events up until 3 weeks following (spanning 6 weeks in total), aggregated weekly, by user. We set the time range as 3 weeks either side of the events based on evidence that most of the psychological changes leading up to self-harm events—particularly suicide-relevant events—that manifest in language typically occur in the preceding 2 weeks (described as the critical period for suicidality^55^), with the most critical changes in the week immediately preceding^35,36^. We thus opted for a 3 week pre-event time frame, with data 3 weeks prior to the event reflective of more general (i.e., “baseline”) language use. The post-event time frame was matched with that of the pre-event period for consistency. Data were aggregated weekly due to the relatively sparse nature of the subsetted dataset. Moreover, previous research has aggregated psychosocial precursors to suicidality data on a weekly level and generated insightful results^44^.

To prepare the subsetted dataset for analysis, overlapping posts coded for recent suicidality or NSSI (i.e., multiple posts disclosing recent suicidality/NSSI in the same 6 week period) by the same user were removed, and multiple posts disclosing recent suicidality/NSSI that were posted on the same day by the same user were merged to be the same event. After removing or merging overlapping posts, 453 cases of recent suicidality and 126 cases of recent NSSI remained. We then refined the dataset further to ensure that all cases had sufficient data for meaningful analysis, as many comprised large portions of missing data (i.e., no or minimal posts in the weeks surrounding the events). Identifying the minimum number of data points necessary to permit the observation of meaningful trends yielded the following analysis inclusion criteria: a minimum of one post made in at least 2/3 of the weeks (i.e., two posts across two separate weeks) prior to the suicidality/NSSI event *and* a minimum of one post made in at least 2/3 of the weeks (i.e., two posts across two separate weeks) following the event. Accordingly, all cases included in subsequent analyses comprise linguistic data (at least one post) from a minimum of 4 of the 6 weeks surrounding the suicidality/NSSI event (i.e., minimum of four posts, comprising at least 25 words each, across four separate weeks surrounding each self-harm event). Applying these criteria resulted in a final subset of 159 cases of recent suicidality from 124 individual users (*N* complete observations = 827) and 43 cases of recent NSSI from 40 individual users (*N* complete observations = 227).

All weekly aggregated data (i.e., averaged linguistic feature scores) were assigned a time point in relation to the suicidality/NSSI events; namely: −3 = 3 weeks before; −2 = 2 weeks before; −1 = 1 week before; 0 = day of event; 1 = 1 week after; 2 = 2 weeks after; 3 = 3 weeks after. Generalised linear mixed models (GLMMs) were performed to examine changes in language in proximity to suicidality and NSSI. GLMMs were selected for the analysis method given that they permit missing data and small sample sizes, and also allow us to nest posts within users and control for random user effects; GLMMs were used over LMMs due all dependant variables (DVs) being non-normally distributed. In all GLMMs, time point was entered as the fixed effect variable (with 6 levels/time points, spanning 3 weeks before the event to 3 weeks after; the day of the event was not included) and the LIWC variables were entered as DVs, with random effects of users controlled for and a log-link transformation applied (due to non-normal data distributions). In cases where significant overall main effects of time were found, post-hoc pairwise comparisons (two-tailed) were conducted to determine precisely when (i.e., between which time points) significant changes in language occurred. These analyses were carried out separately for suicidality and NSSI.

Prior to the main analyses, we conducted descriptive analyses in which we examined changes in the number of posts users made (i.e., posting frequency) to the BPD subreddits in proximity to suicidality and NSSI events via GLMMs (as described above), with number of posts aggregated weekly as the DV. We also investigated changes in the word count (i.e., length) of posts in proximity to suicidality and NSSI events, in which average post word count (aggregated weekly) was entered as the DV. Refer to Supplemental Materials F for results from these descriptive analyses.

We now present the main results from the GLMMs, segregated by psychosocial domain (as in Table 1). Refer to Supplemental Materials G for descriptive statistics and overall fixed effects of time point in proximity to suicidality (Table S7) and NSSI (Table S8) for each of the 16 language variables.

Most of the significant linguistic changes that occurred in proximity to both suicidality and NSSI events were in emotion language. GLMMs revealed no significant fixed effects of time (in proximity to the events) for overall positive or negative emotion words in relation to both suicidality and NSSI events (see Tables S7, S8). However, meaningful changes emerged for several of the specific negative emotion categories.

For suicidality, there was a fixed effect of time (in proximity to suicidality) for anxiety (*F*(5, 821) = 7.85, *p* < 0.001), sadness (*F*(5, 821) = 6.04, *p* < 0.001), and swear words (*F*(5, 821) = 7.60, *p* < 0.001; see Table S7 for descriptive statistics). Anxiety words sharply increased from 3 to 2 weeks before the suicidality event (*M* increase = 0.32, *SE* = 0.06, *t* = 5.59, *p* < 0.001), which remained at a heightened level in the week immediately preceding the event. Although still higher than baseline levels, there was a decrease in anxiety words in the week immediately following the suicidality event when compared to 2 weeks before (*M* decrease = −0.20, *SE* = 0.05, *t* = −3.74, *p* < 0.001), which stayed at the same frequency level 2 weeks after the event. Anxiety words increased again 3 weeks after the event (*M* increase = 0.15, *SE* = 0.06, *t* = 2.51, *p* = 0.012). As for changes in sadness, there was a significant increase in sadness words in the week immediately preceding the suicidality event compared to 3 and 2 weeks before (*M* increase from 2 weeks before = 0.19, *SE* = 0.06, *t* = 3.44, *p* = 0.001). Sadness words dropped back to baseline levels in the week immediately following the suicidality event (*M* decrease = −0.21, *SE* = 0.05, *t* = −3.84, *p* < 0.001), which stayed at the same frequency level 2- and 3 weeks post-event. Swear words also increased in the week immediately preceding the suicidality event compared to 3 and 2 weeks before (*M* increase from 3 weeks before = 0.18, *SE* = 0.05, *t* = 3.26, *p* = 0.001). Use of swear words dropped (back to baseline levels) in the week immediately following the event (*M* decrease = −0.23, *SE* = 0.05, *t* = −4.36, *p* < 0.001), before sharply increasing again 2 weeks post-event (*M* increase = 0.26, *SE* = 0.05, *t* = 4.87, *p* < 0.001). Swear word use decreased again (back to baseline levels) 3 weeks following the suicidality event (*M* decrease = −0.19, *SE* = 0.05, *t* = −3.51, *p* < 0.001). No significant changes in anger words over the weeks surrounding suicidality events were found.

In terms of changes in emotion language surrounding NSSI events, GLMMs revealed a fixed effect of time in proximity to NSSI for anxiety (*F*(5, 221) = 3.27, *p* = 0.007), sadness (*F*(5, 221) = 12.74, *p* < 0.001), and anger words (*F*(5, 221) = 12.33, *p* < 0.001; see Table S8 for descriptive statistics). There were no significant changes in anxiety words across the weeks preceding the NSSI event up until the week immediately following the event. However, anxiety word use significantly dropped 2 weeks following the event compared to all preceding weeks (e.g., *M* decrease from 3 weeks before = −0.26, *SE* = 0.11, *t* = −2.48, *p* = 0.014), which remained at a lower frequency 3 weeks post-event. Use of sadness words increased from 3 to 2 weeks before the NSSI event (*M* increase = 0.36, *SE* = 0.12, *t* = 3.02, *p* = 003), before sharply decreasing the week immediately preceding the event (*M* decrease = −0.54, *SE* = 0.11, *t* = −5.18, *p* < 0.001). Sadness words remained at a lower frequency over the 3 weeks following the NSSI event. There were no changes in anger words across the weeks preceding the NSSI event. However, anger words considerably increased in the week immediately following the event compared to the 3 preceding weeks (e.g., *M* increase from 1 week before = 0.62, *SE* = 0.13, *t* = 4.81, *p* < 0.001), which dropped back down to baseline levels by 2 weeks post-event (*M* decrease = −0.61, *SE* = 0.13, *t* = −4.77, *p* < 0.001). There was no significant overall effect of time in proximity to NSSI for swear words.

**Fig. 1:**
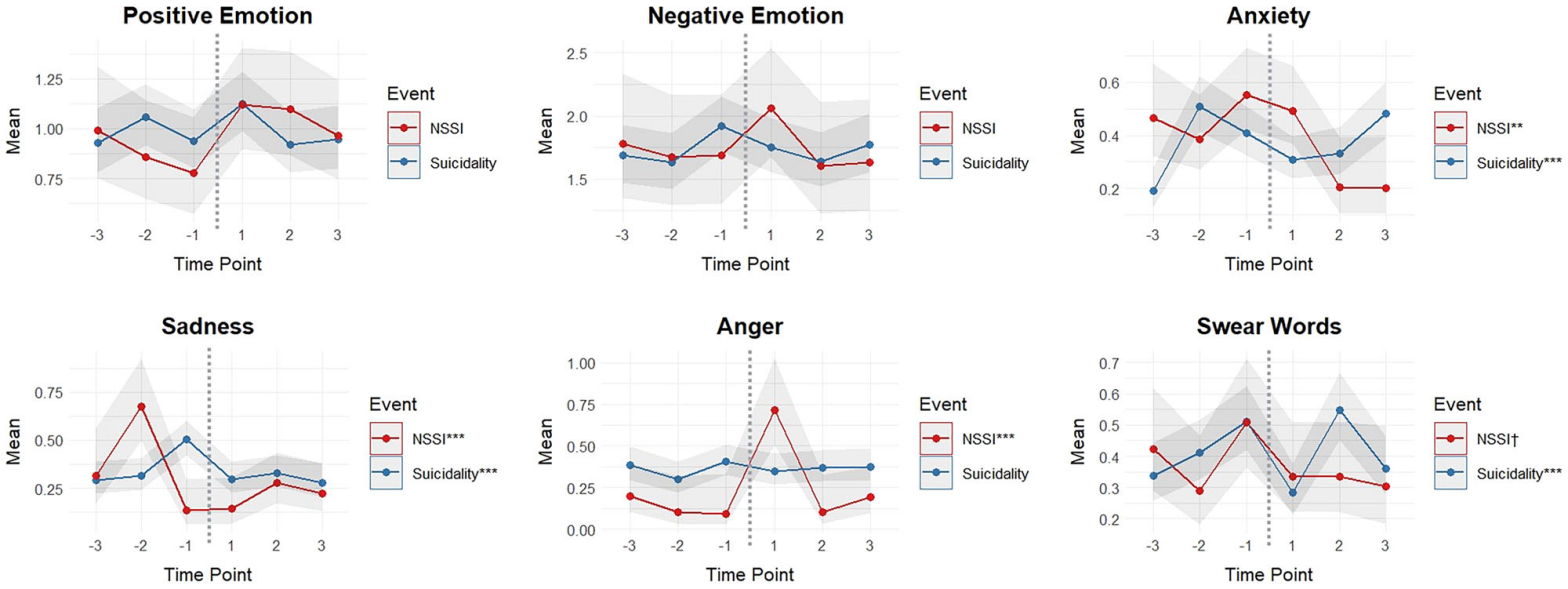
GLMM emotion plots: changes in mean emotion language in proximity to recent suicidality and nonsuicidal self-injury (NSSI) events. The figure shows changes in mean emotion language category scores (derived from LIWC) per week (i.e., aggregated weekly) surrounding suicidality and NSSI events. The dotted lines illustrate the point at which engagement in the event occurred (i.e., time point 0), thus dividing the figures by pre- and post-event. The shaded areas surrounding the means represent the error margins (95% confidence intervals). The means (and confidence intervals) have been estimated from the generalised linear mixed models (GLMMs), and thus are reflective of the repeated measures nature of the data (i.e., person-centered) while also controlling for random user effects. The indicators assigned to the suicidality and NSSI keys show the statistical significance of the overall fixed effects of time in proximity to the events: ***p < 0.001, **p < 0.01, *p < 0.05, ^†^*p* < 0.10. Time point labels: −3 = three weeks before event, −2 = two weeks before event, −1 = one week before event, 1 = one week after event, 2 = two weeks after event, 3 = three weeks after event.

Figure 1 illustrates the weekly changes in emotion language in proximity to both suicidality and NSSI events.

Of the linguistic categories related to social processes (see Table 1), GLMMs for suicidality revealed a fixed effect of time (in proximity to suicidality) for affiliation words (*F*(5, 821) = 4.20, *p* < 0.001) and shehe-words (i.e., third-person singular pronouns; *F*(5, 821) = 3.27, *p* = 0.006; see Table S7 for descriptive statistics). There were no significant changes in affiliation words over the weeks preceding the suicidality event. However, there was an increase in affiliation words in the week immediately following the event compared to 2 weeks and 1 week before (*M* increase from 1 week before = 0.38, *SE* = 0.16, *t* = 2.37, *p* = 0.018), which remained at a slightly heightened frequency 2 weeks post-event. Affiliation word use dropped back down to pre-event frequency levels 3 weeks following the suicidality event (*M* decrease = −0.49, *SE* = 0.17, *t* = −2.89, *p* = 0.004). Shehe-word (i.e., third-person singular pronouns) use significantly decreased in the week immediately preceding the suicidality event compared to 3 and 2 weeks before (*M* decrease from 2 weeks before = −0.45, *SE* = 0.16, *t* = −2.75, *p* = 0.006), which remained at a lower frequency over the 3 weeks following the event. There were no other significant fixed effects of time in proximity to suicidality for any of the other language categories related to social processes.

With regard to NSSI, GLMMs revealed a fixed effect of time in proximity to NSSI for we-words (i.e., first-person plural pronouns; *F*(5, 221) = 12.58, *p* < 0.001) and affiliation words (*F*(5, 221) = 2.60, *p* = 0.026; see Table S8 for descriptive statistics). Specifically, there was a marginally significant decrease in we-words from 3 to 2 weeks before the NSSI event (*M* decrease = −0.16, *SE* = 0.08, *t* = −1.96, *p* = 0.052), which stayed at a similar frequency level in the week immediately preceding the event. We-words sharply increased in the week immediately following the NSSI event compared to all pre-event weeks (e.g., *M* increase from 1 week before = 0.56, *SE* = 0.13, *t* = 4.22, *p* < 0.001), before dropping back down to baseline levels 2 weeks following the event (*M* decrease = −0.34, *SE* = 0.12, *t* = −2.84, *p* = 0.005). Affiliation words were also found to decrease from 3 to 2 weeks before the NSSI event (*M* decrease = −1.03, *SE* = 0.32, *t* = −3.23, *p* = 0.001), remaining at a lower frequency in the week immediately preceding the event. Affiliation word use frequency did not significantly change from 1 week pre-event over the 3 weeks following the NSSI event (but was similar to baseline levels by 1 week post-event). No other fixed effects of time in proximity to NSSI were found for any other social language category.

**Fig. 2:**
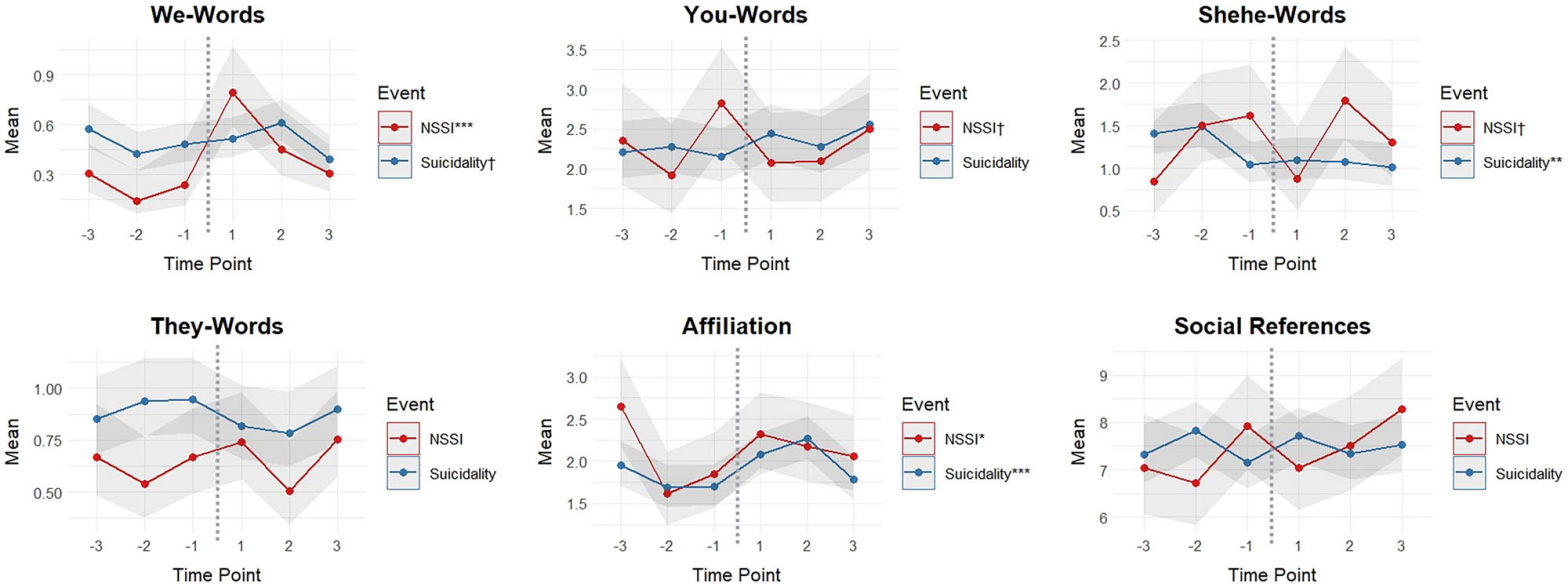
GLMM social plots: changes in mean social language in proximity to recent suicidality and nonsuicidal self-injury (NSSI) events. The figure shows changes in mean social language category scores (derived from LIWC) per week (i.e., aggregated weekly) surrounding suicidality and NSSI events. The dotted lines illustrate the point at which engagement in the event occurred (i.e., time point 0), thus dividing the figures by pre- and post-event. The shaded areas surrounding the means represent the error margins (95% confidence intervals). The means (and confidence intervals) have been estimated from the generalised linear mixed models (GLMMs), and thus are reflective of the repeated measures nature of the data (i.e., person-centered) while also controlling for random user effects. The indicators assigned to the suicidality and NSSI keys show the statistical significance of the overall fixed effects of time in proximity to the events: ***p < 0.001, **p < 0.01, *p < 0.05, ^†^*p* < 0.10. Time point labels: −3 = three weeks before event, −2 = two weeks before event, −1 = one week before event, 1 = one week after event, 2 = two weeks after event, 3 = three weeks after event.

See Fig. 2 for a visual display of weekly changes in social language in proximity to both suicidality and NSSI events.

Regarding self-processes, GLMMs revealed no significant fixed effects of time (in proximity to the events) for I-words or negations in relation to both suicidality and NSSI events (see Tables S7, S8). Specific (mostly statistically non-significant) changes in I-words and negations over the weeks surrounding suicidality and NSSI events can be seen in Figure S4 (Supplemental Materials G).

Likewise, for cognitive processes, no significant fixed effects of time (in proximity to the events) were found for cognitive processing words or absolutist language in relation to both suicidality and NSSI events (see Tables S7, S8). Figure S5 (Supplemental Materials G) displays the linguistic trajectories of these variables in proximity to suicidality and NSSI events.

### RQ2: What features characterise the online BPD community’s interaction with disclosures of suicidality and NSSI?

To address RQ2, we leveraged Reddit community interaction variables from post meta-data; namely, post scores (i.e., the number of “upvotes” [or “likes”] minus “downvotes”) and number of replies to submissions, both of which have been utilised and conceptualised in previous related work as reflecting online community support^34,46^.

We first carried out a general person-level analysis whereby we ran Spearman’s correlations (two-tailed) between users’ mean post scores (averaged from all posts per user; *N* = 992 users), mean number of replies received (for submissions only, averaged per user; *N* = 880 users), and users’ total frequency of posts coded for suicidality (including suicidal ideation/feelings/behaviours) and NSSI (as separate variables; including both past and recent events). To build on these analyses further and more directly address RQ2, we ran a series of independent (two-tailed) *t*-tests, comparing post scores and number of replies received between posts in which suicidality (*N* = 1290 posts) or NSSI (*N* = 678 posts) were disclosed and manually coded posts in which suicidality (*N* = 7816 posts) or NSSI (*N* = 8428 posts) were not disclosed, separately for past and recent events. In all *t*-tests, disclosure of suicidality/NSSI was entered as the IV and post score or number of replies received was entered as the DV. These *t*-tests were carried out on data that had been manually coded/checked for suicidality/NSSI events only (total *N* = 9106 posts).

Regarding the person-level correlation analyses, results revealed a positive association between users’ mean post scores and the frequency of posts disclosing recent suicidality (*r*(990) = 0.10, *p* = 0.002). Associations between mean post scores and frequencies of posts disclosing past suicidality, as well as past and recent NSSI, were all non-significant. For number of replies received (to submissions only), results showed a positive association between users’ mean number of replies received and the frequency of posts disclosing past suicidality (*r*(878) = 0.11, *p* = 0.002). There were no other significant correlation results.

**Fig. 3:**
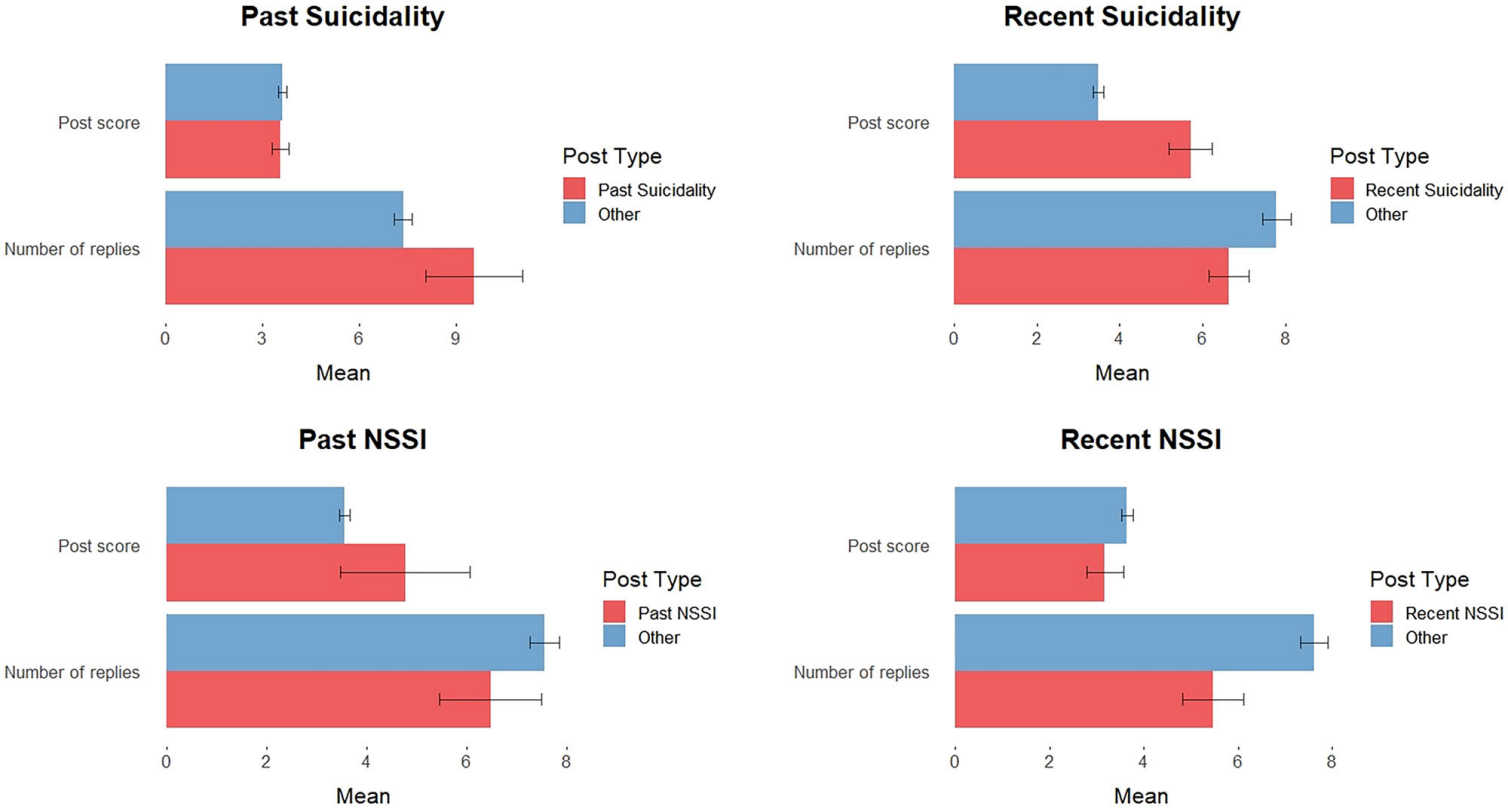
Comparisons of community support generated in self-harm versus non-self-harm posts (*N* = 9106 posts). This figure presents a visual display of the RQ2 *t*-test results, in which post scores and number of replies received were compared between posts disclosing past/recent engagement in suicidality/NSSI versus all other manually coded posts (i.e., posts coded as not disclosing suicidality/NSSI). Post scores reflect the number of upvotes a post receives subtracted by the number of downvotes, thereby reflecting the overall “rating” of the post. Number of replies received are in relation to submissions only (i.e., not responses to comments). The figure is organised by the type of the event disclosed (i.e., suicidality or NSSI) and whether the post disclosed past or recent engagement in the event. Error bars represent the standard errors.

Results from the document/post-level *t*-tests revealed posts disclosing recent suicidality (*N* = 600, *M* = 5.71, *SD* = 12.80) received significantly higher scores (i.e., upvotes) when compared to all other non-suicidality posts manually coded (*N* = 7,816, *M* = 3.48, *SD* = 11.44; *t*(669) = 4.14, *p* < 0.001, *d* = 0.19). There was no significant difference between submissions disclosing versus not disclosing recent suicidality in the number of replies received (*p* = 0.083). As for NSSI, submissions disclosing recent NSSI (*N* = 148, *M* = 5.47, *SD* = 4.76) received significantly fewer replies compared to non-NSSI posts (*N* = 8,428, *M* = 7.62, *SD* = 8.71; *t*(80) = 3.04, *p* = 0.003, *d* = 0.25). However, no differences were found when comparing post scores between posts disclosing recent NSSI versus non-NSSI posts. There were no significant differences in post scores or number of replies received between posts disclosing past engagement in either suicidality or NSSI when compared to non-suicidality/NSSI posts. See Fig. 3 for an illustrative comparison of post scores and number of replies received between posts disclosing suicidality/NSSI versus non-suicidality/NSSI posts.

### RQ3: How might the online BPD community interact with the psychosocial dynamics preceding suicidality and NSSI events to shape the outcome of these events?

As in RQ2, we utilised the post scores and number of replies received (to submissions only) meta-data variables to address this research question. We conducted Spearman’s correlation analyses (two-tailed) between frequencies of key linguistic features and community responses to posts (i.e., post scores and number of replies received), using the full BPD Reddit dataset (i.e., *N* = 66,786 posts). The language variables included in this analysis were those for which significant fixed effects of time were found in proximity to either suicidality or NSSI events in the RQ1 analyses: affiliation words, we-words, shehe words, anxiety, sadness, anger, and swear words. Examining the extent to which these linguistic features correlate with post scores and number of replies received allows insight into how the online BPD community is interacting with the psychosocial dynamics (evident in language) surrounding suicidality and NSSI.

With the exception of affiliation words, all of the tested language variables showed positive correlations with post scores. Specifically, frequencies of we-words (*r*(66,784) = 0.02, *p* < 0.001) and shehe-words (*r*(66,784) = 0.02, *p* < 0.001), as well as anxiety (*r*(66,784) = 0.03, *p* < 0.001), sadness (*r*(66,784) = 0.04, *p* < 0.001), anger (*r*(66,784) = 0.04, *p* < 0.001), and swear words (*r*(66,784) = 0.04, *p* < 0.001), positively correlated with higher post scores. There were no significant correlations between the tested language variables and number of replies received. Full correlation results (i.e., for all variables) are reported in Supplemental Materials H (Table S9).

## Discussion

In the present work, we leveraged modern NLP methods to analyse large online BPD discussion forums to generate better understanding of the psychosocial dynamics of suicidality and NSSI in a critical population (i.e., individuals with BPD), while also examining online community dynamics in relation to self-harm. To our understanding, this is the first study to integrate advanced computational linguistic methods with psychological theory to provide such a far-reaching, large-scale, naturalistic psychological perspective on both suicidality and NSSI in situ, and in a BPD context, where these behaviours are particularly prevalent and problematic. Overall, our findings reveal person-level linguistic features (indicative of psychosocial correlates) of suicidality and NSSI in BPD, validating and extending existing research. The present findings also shed light on the temporal psychosocial dynamics that surround suicidality and NSSI, generating further understanding of why these behaviours occur when they do, in a high-risk population. Further, we evidenced meaningful interactions between the online BPD community and self-harm, in terms of both the disclosure of these behaviours on the platform as well as the temporal psychosocial dynamics that surround these harmful behaviours.

Informatively, the (person-level) linguistic correlates of suicidality and NSSI found in the present work were largely consistent with previous literature^17^. To summarise, our findings uncovered self-focused language, negative emotive language—sadness and anger words in particular—and fewer social references as linguistic features of both suicidality and NSSI, with greater use of negations, swear words, and absolutist language as additional features of suicidality. Interpreting this on a broader psychological level, such features position dysfunctional emotion, social, and self processes as stable psychosocial correlates of both suicidality and NSSI, with maladaptive cognitive processes as an additional correlate of suicidality, at least in high-risk individuals suffering more severe dysfunction. Such knowledge provides greater understanding of why self-harm is so common in individuals with BPD, with these psychosocial features comprising fundamental facets of borderline pathology. Relatedly, our study shines new light on previous findings that have revealed self-harm—specifically NSSI—to be more frequent and severe in individuals with BPD who engage in self-harm compared to those without BPD who engage in self-harm^56^.

In terms of why people engage in self-harm *when* they do, our results illustrating the linguistic trajectories surrounding self-harm provide useful insight in this respect. Notably, consistent with previous NLP suicide/NSSI research, our study revealed the emotion language categories to undergo the most prolific changes in proximity to both suicidality and NSSI events, thus supporting dysfunctional emotion processes as the predominant triggering factor in self-harm^13,14^. It is, however, important to note that the positioning of emotion dysregulation as the main driver of self-harm may be specific to those with BPD, or individuals with more severe psychopathology, on the basis of past findings evidencing more severe depressive symptomatology and emotion dysregulation in individuals with BPD who engaged in NSSI compared to those without BPD who engaged in NSSI^56^.

The emotion language trajectories in proximity to suicidality were generally in alignment with those found in previous NLP research^35^; that is, negative emotive language increased in the weeks preceding suicidality and somewhat decreased again in the week immediately following. Yet, our results also showed increases in swear words to immediately precede suicidality—a novel finding. Greater use of swear words may reflect amplified emotional experiences, of which may therefore precede suicidality, at least among individuals with BPD. The association found (cross-sectionally and temporally) between suicidality and negative emotionality—particularly anger and sadness—is consistent with previous NLP research in non-BPD samples^57,58^. For instance, topic modeling of peer interactions on Reddit’s suicide discussion forum *r/SuicideWatch* revealed “psychological pain” to be the most common topic of suicidality disclosures/posts^58^. The convergence of findings across samples in fact suggests that the connection between suicidality and negative emotionality/psychological pain is strong regardless of the population.

Intriguingly, the emotion language trajectories surrounding NSSI differed considerably from suicidality, and portray a less straightforward picture. In particular, sadness word use increased 2 weeks prior to engagement in NSSI, but drastically decreased in the week immediately preceding. Combined with generally high anxiety (reflected by high frequencies of anxiety words), this decrease in sadness (words) may potentially reflect a period of “emotional numbness” or dissociation—an evidenced precursor to NSSI in people with BPD^21^. Thus, our findings indicate support to more traditional research evidencing dissociation as a precursor to NSSI in individuals with BPD, which differs from findings in other (non-BPD) contexts showing heightened negative emotion to precede NSSI^40^. As with suicidality, there were no changes in positive emotion words in proximity to NSSI, implying that positive emotion words are not reliable markers of self-harm in individuals with BPD. Such discrepancies appear to indicate some distinguishing emotion-related precursors to self-harm, particularly NSSI, between people with BPD, or high-risk individuals suffering severe dysfunction, and the broader population who engage in self-harm. This notion is in alignment with past work that has demonstrated key differences in clinical characteristics of NSSI in individuals with BPD compared to those without BPD^56^.

Other striking patterns of results surround the emotion language changes that occurred following NSSI. Namely, anxiety word use considerably decreased 2 weeks following NSSI, supporting the notion that NSSI serves affect regulation functions (i.e., reducing anxiety, in this case)^14^. However, despite decreases in anxiety, anger words sharply increased immediately following the NSSI event. Interpreting the findings together, while individuals may engage in NSSI in an attempt to regulate their emotions (e.g., anxiety) and relieve dissociation/emotional numbness, engagement in NSSI in fact appears to generate further emotion dysregulation—particularly in the form of heightened anger—indicating majorly maladaptive affect regulation–self-harm cycles.

In addition to emotion language trajectories, other insightful findings emerged when examining changes in social language in proximity to self-harm. In alignment with previous findings^34^, other-focused language (i.e., third-person singular pronouns) decreased in the week immediately preceding suicidality, whereas affiliation words (indicating social connectedness) increased following suicidality events. Likewise, linguistic changes in proximity to NSSI displayed the same trend, with linguistic indicators of social connectedness decreasing leading up to NSSI and increasing immediately following. These findings provide support to the notion that self-harm, to some extent, serves social functions^15,16,31,32^, and align with findings from more traditional research evidencing social dysfunction as a key precursor to both NSSI^42^ and suicidality^41^ in people with BPD. Likewise, our findings are also in alignment with recent NLP research carried out using a non-BPD sample, which uncovered the topic of “relationship stress”—comprising a focus on recent disruptions in close interpersonal relationships—to be the second largest topic characterising suicidality disclosures on the *r/SuicideWatch* Reddit discussion forum^58^, highlighting the universally significant role of relationship issues in the evolution of self-harm. Moreover, in terms of BPD theory specifically, the present findings have implications for the interpersonal hypersensitivity model of BPD, which proposes that social dysfunction, and specifically heightened sensitivity to interpersonal stressors (e.g., negative biases about others; fears of abandonment), lie at the core of dysfunction in BPD^59^. Indeed, our study has provided some support for this model in indicating that internalised forms of social dysfunction evident in natural language (i.e., social-cognitive dysfunction) are central in the development and manifestation of harmful thoughts and behaviours (i.e., self-harm) in BPD.

To summarise and integrate the linguistic features found to precede self-harm, suicidality was preceded by co-occurring heightened anxiety words and decreased other-focused language (i.e., third-person singular pronouns), shortly followed by heightened sadness words and swear words, as well as posting more frequently to the BPD forum (refer to Supplemental Materials F). As for NSSI, this was preceded by heightened sadness words and decreased socially connected language (i.e., we-words and affiliation words), along with posting less frequently to the online BPD forum (see Supplemental Materials F), immediately followed by a sharp drop in sadness word use. Provided such linguistic trajectories receive validation in different samples, this knowledge could prove crucial regarding the anticipation and prevention of self-harm. More broadly, these patterns highlight how dysfunctional emotion and social processes co-occur and most probably interact with one another in triggering both suicidality and NSSI in high-risk populations (i.e., individuals with BPD), illuminating a pivotal area for future research.

To gain insight into possible effects of online BPD community dynamics on self-harm—acknowledging contextual factors^45^—we first examined how the online community interacted with disclosures of self-harm (RQ2). Results revealed disclosures of recent suicidality (including suicidal ideation/feelings/behaviours) to be associated with more community support (relative to non-suicidality posts), primarily in the form of upvotes (or post scores), which may partially explain the increase in socially connected language following suicidality disclosures. Disclosures of NSSI, by contrast, did not generate greater community support. In fact, posts disclosing recent engagement in NSSI received fewer replies, on average, when compared to non-NSSI posts.

Findings from the RQ2 analyses generate multiple potential hypotheses. One possible hypothesis is that the elevated support provided by the online community in response to suicidality disclosures may help to prevent future suicidality through providing much needed social support and a sense of belonging, as implied in previous work^34^. Alternatively, it could be that greater community support, in the form of upvotes, inadvertently reinforces (disclosures of) suicidality, further driving suicidality. Indeed, empirical support for this hypothesis can be seen from fMRI research showing how “likes” (or upvotes) on social media posts tap into the reward pathway in the brain^60^. It is therefore possible that the relatively greater numbers of upvotes given to posts disclosing suicidality, although unintentional, could reinforce suicidality.

Valuably, the RQ3 analyses—whereby we examined interactions between the online community and the temporal linguistic dynamics that surround self-harm—shed further light on how community dynamics may drive or hamper engagement in self-harm. Consistent with previous work^46^, and with the interpersonal hypersensitivity model of BPD^59^, our results illustrated that the online BPD communities are typically providing more upvotes to posts when they display more socially connected/orientated language (i.e., we-words and shehe-words). Notably, this finding is also consistent with previous research finding “relationship/loss support” to be one of the most helpful topics (measured based on the number of upvotes a comment received) in response to suicidality disclosure posts^58^. However, the BPD communities also scored posts higher when they displayed more negative emotive and hostile language (i.e., anxiety, sadness, anger, and swear words), directly contradicting previous findings^46^. Such discrepancies could be explained by differences in the community population studied (i.e., BPD vs. general mental health support communities). Indeed, distinguishing features of BPD include intense negative emotionality and rumination^61^, with evidence to suggest that individuals with BPD engage in negative rumination, particularly anger rumination, as a (maladaptive) emotion regulation strategy, with prolonged thoughts about anger/hostility allowing for attention to be directed toward external (as opposed to internal) sources of distress^62^. Further, provocation-focused rumination has been found to be associated with neural activity in reward networks in individuals with BPD^63^. Hence, the seemingly rewarding (yet problematic) nature of pre-occupation with negative emotionality in those with BPD could explain their inclination to like/upvote posts expressing high levels of negativity and hostility, with these posts (and the act of rewarding them) potentially stimulating feelings of empowerment and validation (i.e., negative emotions are justified).

Interpreting our results together, the influence of online BPD communities on suicidality and NSSI appears to be mixed. From one perspective, the BPD communities appear to hamper engagement in self-harm by implicitly discouraging expressions of social-disconnectedness in posts (through providing more support to posts displaying greater social-connectedness)—a precursor to both suicidality and NSSI. However, the communities also appear to have a potential driving effect on engagement in self-harm through rewarding (via more upvotes) displays of emotion dysregulation and hostility in posts, which precedes self-harm; an effect that is seemingly specific to BPD communities. Yet, such interpretations remain speculative at present, as causal effects have not been directly tested; this is a crucial area for future research.

Despite the many strengths of the current dataset—including a large, naturalistic sample of individuals with self-identified BPD—it is inherently accompanied by biases associated with the use of Reddit data that should be acknowledged. First, it is possible that our findings may be specific to disclosures of self-harm on Reddit and may not be generalisable to language more broadly (e.g., text messages). Second, our sample comprised individuals with self-identified BPD who, at least at one point, were frequent users of Reddit (specifically the BPD subreddits), indicating that our sample may not be representative of the broader population of individuals with BPD of whom do not (frequently) use (or have never used) Reddit. Third, our BPD classification relied upon users’ self-identified BPD statements, and thus was not clinically verified. Finally, we relied on users of the BPD platform being honest in their disclosures of suicidality and NSSI; disclosures of self-harm were taken as the ground truth. Yet, it is possible that users might occasionally not have been truthful in their disclosures. However, given that Reddit is an anonymous platform, and that the BPD subreddits in particular encourage open, honest, and supportive discussion, it would only be in users’ best interests to be open and truthful in their posts.

Our research is further limited by the fact that we did not have a comparison community comprising individuals who engage in self-harm who do not have BPD to allow us to compare our findings, and determine the generalisability of our findings, outside the context of BPD. Thus, our findings may be exclusive to high-risk individuals suffering more severe dysfunction, or people with BPD more exclusively, which should be taken into account when interpreting our findings and the implications drawn.

In terms of analytic limitations, the exact timing of the suicidality/NSSI events was not always clear from users’ posts. Thus, when posts were recorded as an occurrence of “recent engagement in suicidality/NSSI”, this sometimes will have been several days out from the exact day of the occurrence. This limitation was generally not an issue for the coding of recent suicidality events, as posts disclosing recent suicidality predominately comprised users disclosing feeling suicidal in the moment of writing the post (e.g., “I feel so suicidal”), or more generally on the same day of writing (e.g., “I have been feeling suicidal all day”). However, disclosures of recent engagement in NSSI were sometimes less precise regarding the exact occurrence of the event. Nevertheless, the coding procedure was conservative, and posts were only coded as recent engagement if it was clear that the behaviour/event occurred recently.

Novel contributions from the present research span multiple disciplines, yielding both theoretical and practical insights. In terms of theoretical implications, from a clinical psychology standpoint, the present work contributes to suicide and NSSI theory by providing further insight—utilising naturalistic modern methods—into the psychosocial correlates and temporal dynamics that surround suicidality and NSSI in high-risk individuals. In the realm of personality and individual differences, the present work contributes to personality (disorder) theory in that our findings enhance understanding of the nature of self-harm in the context of BPD. Relatedly, our findings also have implications for BPD-specific theories, such as the interpersonal hypersensitivity model of BPD^59^. More generally, our study provides a naturalistic behavioural approach to understanding behavioural dysregulation in personality dysfunction, of which is less prone to bias than traditional methods. From a computational social science perspective, this work enriches the application of NLP to investigate self-harm in online forums, with one of the most comprehensive data annotation schemes undertaken in this domain, contributing to the field of clinical NLP. Finally, in the domain of cyberpsychology, our work generated initial insight into how self-harm may be influenced by online (BPD) support communities, sparking critical hypotheses to be tested in future research.

Regarding practical implications, first, our findings provided insight into the psychosocial features of suicidality and NSSI in the context of BPD. Such insight is of clinical value as, pending validation, these psychosocial features could be monitored by clinicians to identify at-risk individuals likely to engage in these harmful behaviours at concerning rates, upon which appropriate psychological interventions could be provided. More valuably, the present work generated further insight into the temporal psychosocial dynamics that precede (and follow) suicidality and NSSI, which is vital knowledge for clinicians with respect to anticipating when, precisely, high-risk individuals are likely to engage in such harmful behaviours, subsequently helping to lessen the likelihood of self-harm occurring. Put simply, our study has uncovered key linguistic precedents to self-harm in those at high risk, laying the groundwork for subsequent predictive models that could aid in early intervention. To give a specific practical example, our findings showing changes in social language leading up to self-harm could be integrated into interpersonal hypersensitivity models used in clinical treatment for BPD^64^, by monitoring linguistic indicators of social (dys)function (e.g., frequency of we-words) in the language of those undergoing treatment. On a broader level, our findings highlight periods of heightened emotion dysregulation and social dysfunction as the predominant precedents to self-harm, illuminating the most critical areas to be targeted through therapeutic intervention.

To summarise, in the present work, we leveraged modern natural language processing methods to investigate the psychosocial dynamics of suicidality and NSSI in the context of BPD (a high-risk population with regard to self-harm), while also examining online BPD community dynamics in relation to self-harm. To our knowledge, this is the first study to have provided such a far-reaching, large-scale, naturalistic perspective on both suicidality and NSSI in situ, while also incorporating meaningful psychological theoretical perspective. By integrating advanced methods and theories, our findings provide a nuanced understanding of suicidality and NSSI in high-risk individuals, with implications for clinical practice, clinical and personality theory, and computational linguistic research. Our work marks a significant step toward a holistic comprehension of self-harm phenomena, fostering collaboration across disciplines to address this critical public health concern.

## Supplementary information

Supplementary information

## Data Availability

The dataset generated and analysed during the current study is not publicly available due to ethical restrictions which prevent the public sharing of our data. Although Reddit data is in the public domain, the nature of our manual coding for BPD identification, demographic characteristics, and self-harm behaviours/events makes this dataset particularly sensitive. The BPD and Behaviour Reddit Dataset (BBRD^48^)—a broader dataset comprising data used in this study (i.e., data spanning 2011–2019)—is available from the corresponding author, or through Lancaster University Research Data Management services (10.17635/lancaster/researchdata/697), on reasonable request (i.e., researchers who meet the criteria for access to confidential data by signing a data usage agreement with Lancaster University).
