# Supplementary material for: Psychosocial dynamics of suicidality and nonsuicidal self-injury: a digital linguistic perspective
Source: Npj Ment Health Res. 2025 Jul 8;4:28. doi: 10.1038/s44184-025-00142-w (PMC12238424; doi:10.1038/s44184-025-00142-w)
Supplement: Supplementary file 1 — Supplementary information [file 44184_2025_142_MOESM1_ESM.docx]

**Supplemental Material for:**

**Psychosocial Dynamics of Suicidality and Nonsuicidal Self-Injury:**

**A Digital Linguistic Perspective**

Charlotte Entwistle^1,2^, Katie Hoemann^3,4^, Sophie J. Nightingale^1^, Ryan L. Boyd^5^

^1^ Lancaster University, United Kingdom

^2^ University of Liverpool, United Kingdom

^3^ University of Kansas, USA

^4^ KU Leuven, Belgium

^5^ University of Texas at Dallas, USA

**Author Notes**

Charlotte Entwistle
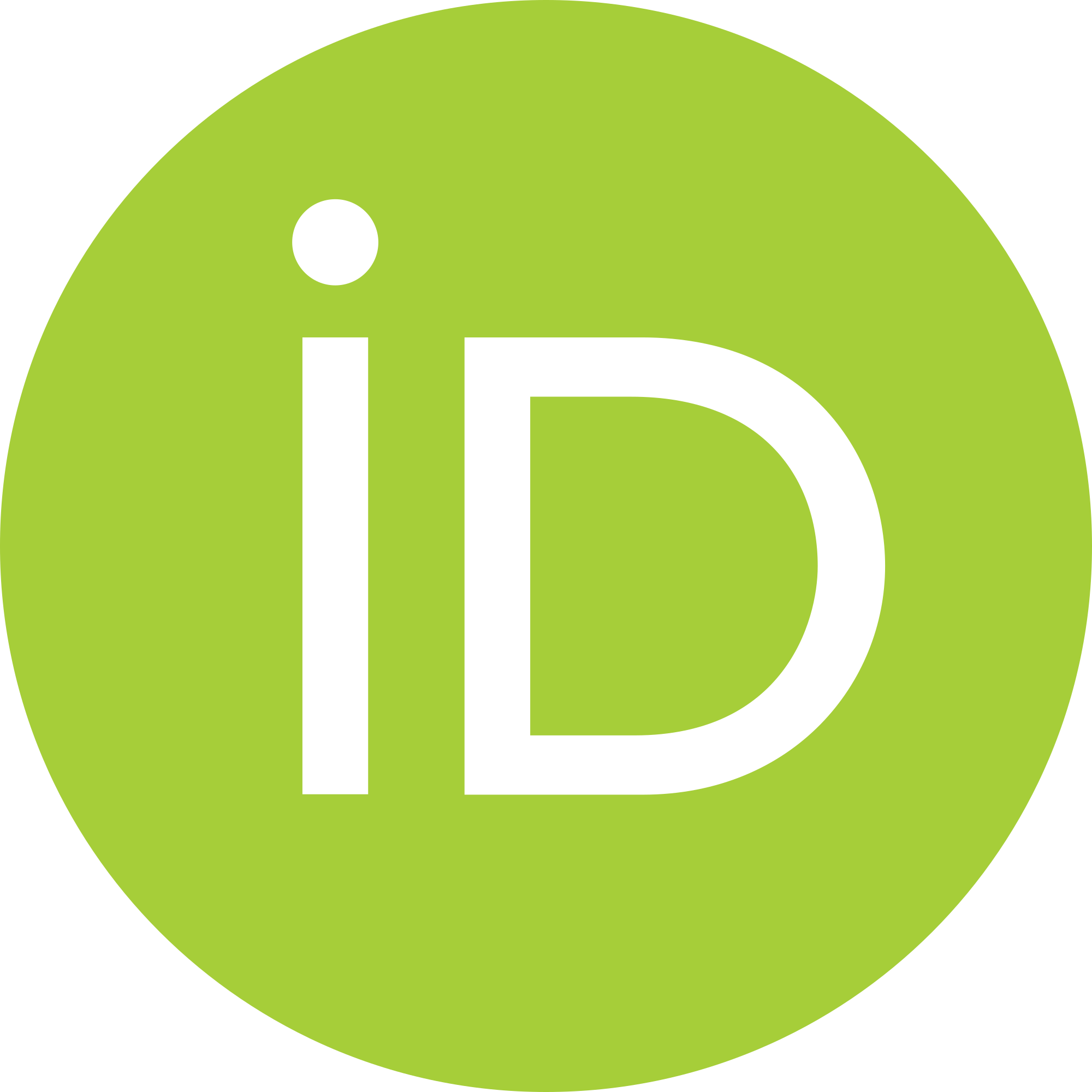
 https://orcid.org/0000-0002-2739-2644

Katie Hoemann
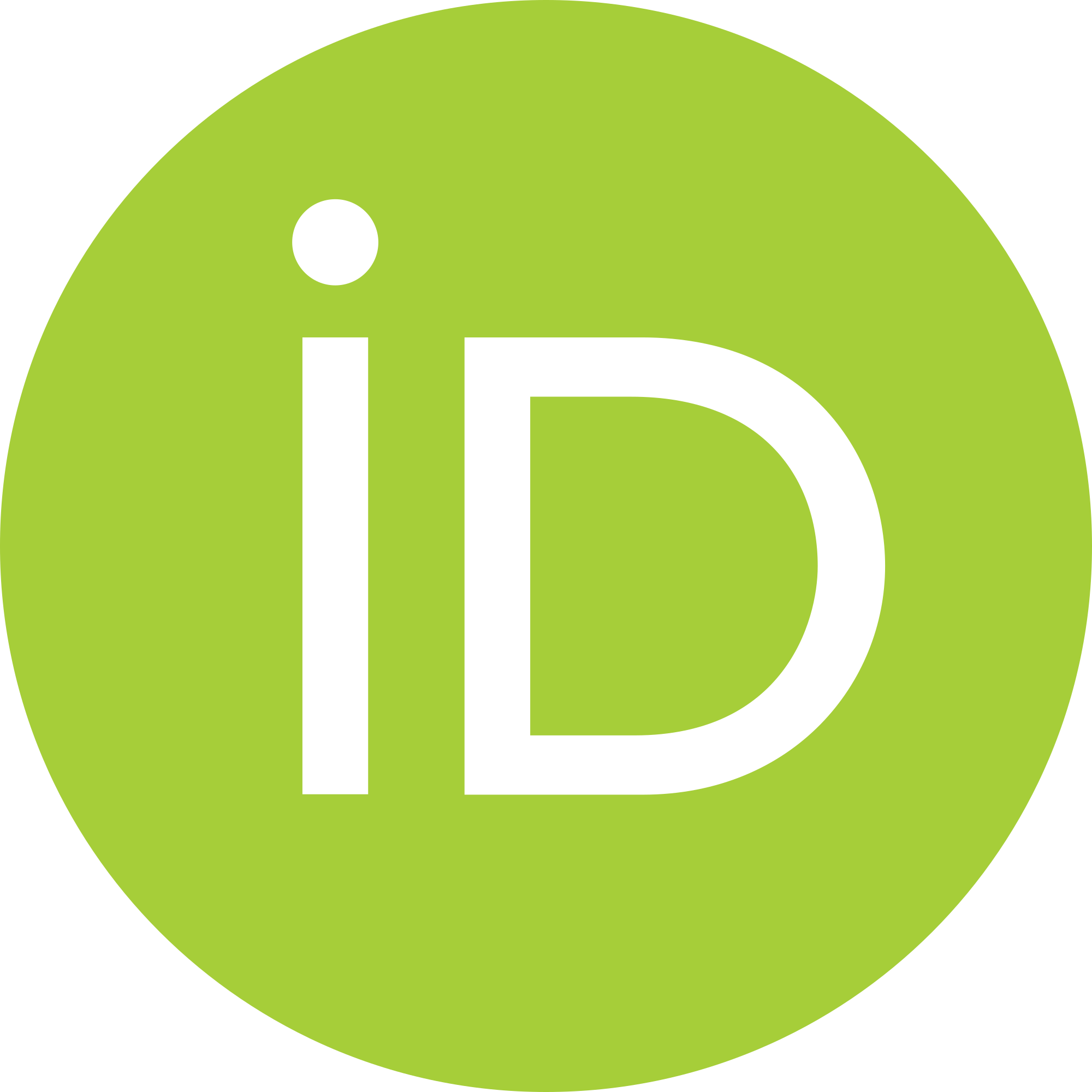
 https://orcid.org/0000-0002-9938-7676

Sophie J. Nightingale
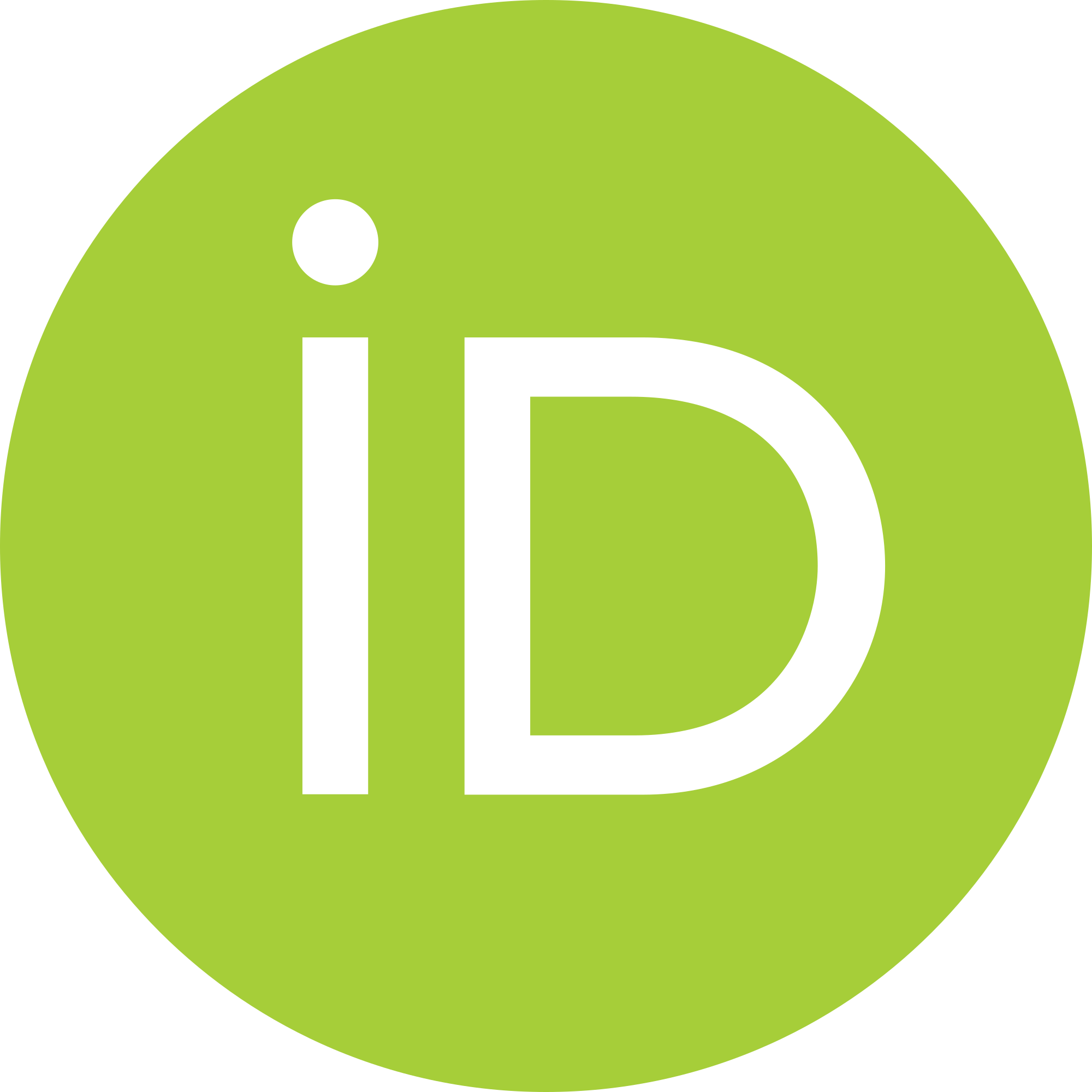
 https://orcid.org/0000-0002-6779-9203

Ryan L. Boyd
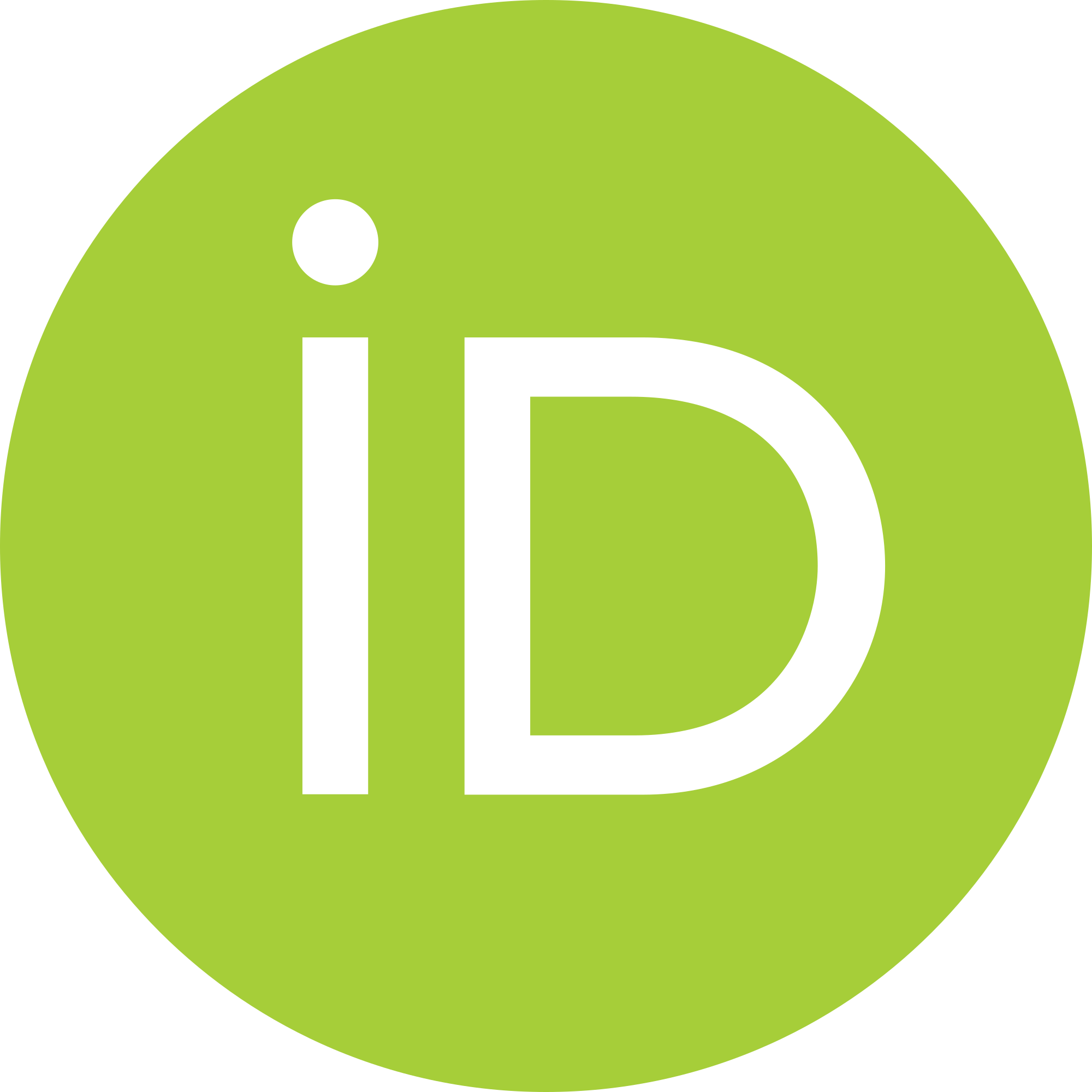
 https://orcid.org/0000-0002-1876-6050

**Supplemental Materials A:**

**Borderline Personality Disorder Reddit Dataset – Self-Harm Coding Guidance**

**Suicidality**

- Broad definition: suicidality broadly reflects risk of suicide, indicated by suicidal ideation/feelings (i.e., thoughts/feelings about suicide) and suicidal behaviours (i.e., preparing and attempting suicide)
- Code for the presence of suicidal thoughts/feelings (i.e., ideation) and suicidal behaviour (i.e., attempts)
- Referencing thoughts/feelings/wishes about wanting to die or to not be alive (in any form) should be coded under suicidal ideation
- Suicidal behaviour will primarily consist of an attempt at suicide (whether or not there is intention to die), which could be achieved by any means; behavioural preparation for suicide can also come under suicidal behaviour
- If someone says or indicates that they were suicidal in the past/have been suicidal before (or attempted suicide/engaged in suicidal behaviour in the past), but not necessarily that they are suicidal currently, code this under past occurrence
- More importantly, if someone makes it clear that they feel suicidal currently or have made a suicide attempt/engaged in suicidal behaviour recently (within the last week), then code this as a recent occurrence – note that they don’t necessarily have to specify when precisely, as long as it is very clear that it occurred recently or is how they are currently feeling
- If it is unclear when the suicidal ideation/behaviour occurred, and there is no evidence to suggest it is recent enough to be within the last week, code as a past occurrence
- If someone indicates they have recently engaged in suicidal behaviour (e.g., attempted suicide) and also currently feel suicidal (at the time of posting), code for both recent suicidal ideation and behaviour (same for past occurrences of ideation and behaviour present in the same post)
- Examples of disclosures of recent/current suicidal ideation/feelings that should be coded for include: “I just don’t want to be alive anymore”; “My suicidal thoughts are getting stronger every day”; “I wish I was dead”
- Examples of disclosures of recent suicidal behaviour/attempts that should be coded for include: “Just a couple of days ago I tried to take my own life”; “I took an overdose yesterday”; “I tried to end it all earlier this week”

**Deliberate self-harm/nonsuicidal self-injury**

- Broad definition: an intentional act of causing oneself physical harm/injury without suicidal intent; including explicit self-harm behaviours, such as cutting, burning, and hitting oneself, as well as other, less explicit forms of self-harm (i.e., indirect self-harm), such as substance abuse and otherwise risky behaviours (e.g., dangerous driving; unsafe sex).
- Self-harming behaviour can take various forms – code for anything that represents intentional behaviour that could/is designed to harm oneself
- Common examples include cutting, hitting/scratching oneself, burning oneself, (intentionally self-harmful) excessive alcohol consumption/substance abuse, binge-eating (to intentionally cause oneself harm), purposely not eating (intentionally starving oneself as a form of self-harm), and other forms of potential self-harm (e.g., purposively dangerous driving or unsafe sex, with the intention of self-harm)
- Only code actual engagement in self-harm behaviour (i.e., the action of harming oneself) under engagement in self-harm
- Current thoughts about self-harm or the feeling of “wanting to self-harm” can be coded for under urge to self-harm
- If it is unclear when the self-harm occurred, and doesn’t appear recent enough to be within the last week, code as a past occurrence
- Only code as a recent occurrence of engagement in self-harm if the post strongly indicates that this occurred within the same week
- Examples of disclosures of recent self-harm that should be coded for include: “I can’t stop self-harming”; “I relapsed with my self-harm on Monday”; “I cut myself last night”
- Examples of having an urge to self-harm that should be coded for include: “I have an urge to self-harm again”; “thinking about this makes me want to self-harm”; “I’m struggling to stop thinking about harming myself”

**Supplemental Materials B:**

**Borderline Personality Disorder Reddit Community Inter-Rater Coding Agreements**

**Table S1**

*Borderline Personality Disorder Subreddit Coding Agreement Percentages*

| Coded Variable | Agreement Percentage |
| --- | --- |
| BPD classification | 94.77 |
| Demographics | 95.67 |
| Age | 97.96 |
| Gender | 92.54 |
| Ethnicity | 98.82 |
| Country of residence | 96.24 |
| Relationship status | 92.60 |
| Religion | 98.15 |
| Behavioural categories | 93.91 |
| Suicidality | 91.52 |
| Nonsuicidal self-injury | 94.14 |

**Supplemental Materials C:**

**BPD Reddit Sample Posting Behaviour – Descriptive Illustrations**

**Figure S1**

*Frequency of Users’ (N = 992) Posts to BPD Subreddits Over Time*


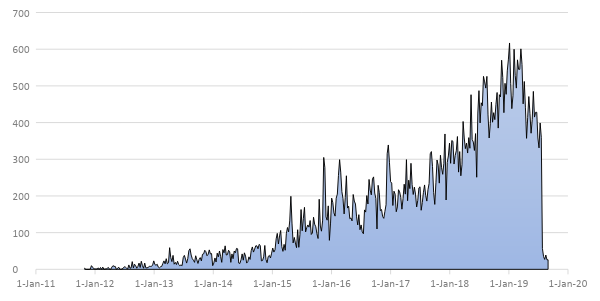


**Figure S2**

*Histogram of Frequency of Days Between Users’ (N = 992) First and Last Posts to the BPD Subreddits*


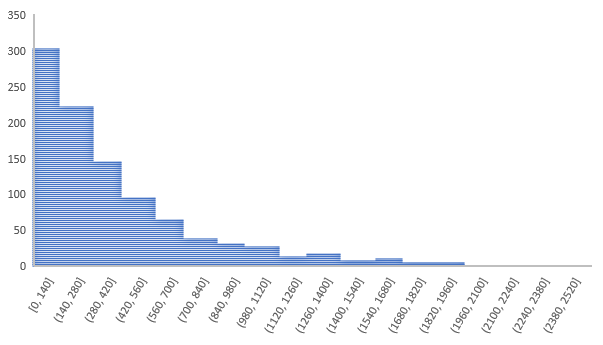


**Supplemental Materials D:**

**BPD Reddit Sample Self-Harm Coding Frequencies**

**Table S2**

*Frequencies of Suicidality and Nonsuicidal Self-Injury (NSSI) Events Manually Coded (N = 992 Users)*

| Behaviour/Event | *N* | % Of Behavioural Category | % Of Coded Posts  (*n* = 9,106) | % Of Total Posts  (*n* = 66,786) |
| --- | --- | --- | --- | --- |
| Suicidality | 1,290 |  | 14.17 | 1.93 |
| Past suicide behaviour | 225 | 17.44 | 2.47 | 0.34 |
| Past suicidal ideation | 465 | 36.05 | 5.11 | 0.70 |
| Recent suicide behaviour | 23 | 1.78 | 0.25 | 0.03 |
| Recent suicidal ideation | 577 | 44.73 | 6.34 | 0.86 |
| Nonsuicidal self-injury | 678 |  | 7.45 | 1.02 |
| Past NSSI | 504 | 74.34 | 5.53 | 0.75 |
| Recent NSSI | 148 | 21.83 | 1.63 | 0.22 |
| Urge for NSSI | 26 | 3.83 | 0.29 | 0.04 |

*Note.* The “*N*” column reflects the total number of suicidality and NSSI events coded.

**Supplemental Materials E:**

**RQ1 Variations of Correlation Analyses – Results Tables**

**Table S3**

*Spearman’s Rho Correlations Between Mean Language Variable Scores and Suicidality and Nonsuicidal Self-Injury (NSSI) Frequencies, with Outliers Removed (N = 992 Users)*

| LIWC Variable | Past Suicidality | Recent Suicidality | Past NSSI | Recent NSSI |
| --- | --- | --- | --- | --- |
| I | .02 | .16*** | .06^†^ | .11*** |
| Negations | .03 | .07* | .04 | .02 |
| Positive emotion | .04 | .05 | .04 | -.04 |
| Negative emotion | .06^†^ | .14*** | .05 | .12*** |
| Anxiety | .07* | .05 | .07* | .05 |
| Sadness | .08* | .12*** | .08* | .09** |
| Anger | .03 | .13*** | .04 | .09** |
| Swear | .04 | .10** | .03 | .03 |
| We | .02 | -.03 | .01 | -.02 |
| You | -.02 | -.06^†^ | -.02 | -.06^†^ |
| Shehe | -.02 | -.03 | .00 | .03 |
| They | .02 | .03 | .00 | .02 |
| Affiliation | -.01 | -.04 | -.04 | .04 |
| Social references | -.03 | -.06^†^ | -.06^†^ | -.06^†^ |
| Cognitive processes | -.08** | -.01 | -.05 | -.10** |
| Absolutism | .04 | .11*** | .00 | .01 |

****p* < .001, ***p* < .01, **p* < .05, ^†^*p* < .10.

*Note.* All tests are two-tailed. Language variable scores reflect users’ mean LIWC22 category scores from the BPD subreddits, excluding posts coded for NSSI or suicidality of any nature. Mean language scores were correlated with users’ overall frequency of suicidality/NSSI, after removing outliers. One outlier was removed for each of the following measures: past suicidality, recent suicidality, and past NSSI.

| LIWC Variable | Past Suicidality | Recent Suicidality | Past NSSI | Recent NSSI |
| --- | --- | --- | --- | --- |
| I | .12*** | .18*** | .12*** | .16*** |
| Negations | .01 | .11*** | .02 | .02 |
| Positive emotion | -.00 | .00 | -.00 | -.03 |
| Negative emotion | .04 | .10** | .03 | .09** |
| Anxiety | .03 | .00 | .02 | .03 |
| Sadness | .05^†^ | .07* | .02 | .08* |
| Anger | .02 | .10** | .04 | .06^†^ |
| Swear | .03 | .13*** | .04 | .06^†^ |
| We | -.05 | -.05 | -.04 | -.04 |
| You | -.12*** | -.10** | -.08** | -.09** |
| Shehe | -.03 | -.05 | -.02 | .02 |
| They | -.02 | .01 | -.02 | -.02 |
| Affiliation | -.02 | -.03 | -.02 | .04 |
| Social references | -.11*** | -.09** | -.08** | -.07* |
| Cognitive processes | -.06^†^ | -.03 | -.04 | -.11*** |
| Absolutism | .02 | .12*** | .02 | -.01 |

**Table S4**

*Spearman’s Rho Correlations Between Mean Language Variable Scores and Suicidality and Nonsuicidal Self-Injury (NSSI) Frequencies, Controlling for Users’ Total Number of Posts (N = 992 Users)*

****p* < .001, ***p* < .01, **p* < .05, ^†^*p* < .10.

*Note.* All tests are two-tailed. Language variable scores reflect users’ mean LIWC22 category scores from the BPD subreddits, excluding posts coded for NSSI or suicidality of any nature (past or recent). Mean language scores were correlated with users’ overall frequency of suicidality/NSSI disclosures, while controlling for users’ overall number of posts.

**Supplemental Materials F:**

**RQ1 Subsetted Dataset – Descriptive Analyses**

As part of RQ1, we conducted descriptive statistical analyses on the subsetted dataset in which we examined changes in the number of posts users made (i.e., posting frequency) to the BPD subreddits in proximity to suicidality and NSSI events via GLMMs (as described in the main manuscript), with number of posts aggregated weekly entered as the DV. These analyses were carried out using the full subsetted dataset prior to further data refinement (i.e., 453 cases of recent suicidality and 126 cases of recent NSSI), given that cases comprising weeks/time points with 0 posts are not classified as missing data in this analysis (i.e., there is no missing data). See Table S5 for the estimated means and standard errors for number of posts made per week surrounding suicidality and NSSI events.

The GLMM conducted for suicidality revealed a significant, large fixed effect of time point (in proximity to suicidality) on posting frequency (*F*(5, 2712) = 31.03, *p* <.001). This effect resulted from a significant increase in the number of posts made in the week immediately preceding the suicidality event compared to 3 and 2 weeks before (*M* increase from 2-weeks pre-event = 1.29, *SE* = 0.21, *t* = 6.24, *p* <.001). Posting frequency remained heightened 1 week after the suicidality event, but significantly decreased by 2-weeks post-event (*M* decrease = -1.19, *SE* = 0.21, *t* = -5.79, *p* <.001); decreasing further (and returning to baseline levels) by 3-weeks post-event (*M* decrease = -0.53, *SE* = 0.20, *t* = -2.70, *p* = .007).

There was also a significant overall fixed effect of time point in proximity to NSSI on posting frequency (*F*(5, 750) = 14.34, *p* <.001). Regarding specific changes, there was a drop in posting frequency 2 weeks preceding the NSSI event compared to 3 weeks before (*M* decrease = -1.00, *SE* = 0.40, *t* = -2.51, *p* = .012), which returned to baseline levels (i.e., 3 weeks pre-event) by the week immediately preceding the event. Further, there was a significant increase in posting frequency in the week immediately following the NSSI event compared to each of the 3 weeks preceding the event (*M* increase from 1 week before = 1.57, *SE* = 0.41, *t* = 3.80, *p* <.001), which sharply decreased again by 2-weeks post-event (*M* decrease = -2.44, *SE* = 0.45, *t* = -5.43, *p* < .001) and remained at a similar level 3-weeks post-event.

In addition to examining changes in posting frequency, we also investigated changes in the word count (i.e., length) of posts made to the BPD subreddits in proximity to suicidality and NSSI events. For this analysis, average post word count (aggregated weekly) was entered as the DV. We used the refined version of the subsetted dataset for this analysis (i.e., 159 cases for suicidality and 43 cases for NSSI), as used with all other linguistic variables, to ensure that all cases have sufficient, high-quality data. See Table S6 for the estimated means and standard errors for post word count for each week surrounding suicidality and NSSI events.

GLMMs revealed no statistically significant overall fixed effect of time point in proximity to suicidality on post length (*F*(5, 821) = 2.19, *p* = .054). However, a significant fixed effect of time point in proximity to NSSI on post length was evidenced (*F*(5, 221) = 39.75, *p* <.001). This effect largely stemmed from considerably shorter post lengths in all weeks surrounding the NSSI event when compared to 3-weeks pre-event (e.g., *M* decrease from 3- to 2-weeks pre-event = -101.53, *SE* = 12.59, *t* = -8.07, *p* <.001). Yet, post length was found to significantly increase from 2 weeks to 1 week before the NSSI event (*M* increase = 31.48, *SE* = 9.07, *t* = 3.47, *p* = .001), which stayed around the same level in the week immediately following the event. Post length significantly decreased again 3 weeks after the NSSI event (compared to 1-week post-event; *M* decrease = -23.42, *SE* = 9.18, *t* = -2.55, *p* = .011).

See Figure S3 for a visual display of weekly changes in posting frequency and post length (i.e., word count) in proximity to suicidality and NSSI events.

**Table S5**

*Estimated Means and Standard Errors (SE) for Number of Posts Per Week in Proximity to Suicidality and Nonsuicidal Self-Injury (NSSI) Events*

|  | Suicidality (N cases = 453; N observations = 2,718) | | NSSI (N cases = 126; N observations = 756) | |
| --- | --- | --- | --- | --- |
| Time | Mean | *SE* | Mean | *SE* |
| 3 weeks before | 2.10 | 0.18 | 3.35 | 0.42 |
| 2 weeks before | 2.39 | 0.19 | 2.36 | 0.35 |
| 1 week before | 3.68 | 0.23 | 3.36 | 0.42 |
| 1 week after | 3.90 | 0.24 | 4.93 | 0.54 |
| 2 weeks after | 2.71 | 0.20 | 2.49 | 0.36 |
| 3 weeks after | 2.18 | 0.18 | 2.35 | 0.35 |

*Note.* The means and standard errors presented here have been estimated from the GLMMs, and thus are in accordance with the repeated measures nature of the data (i.e., person-centered) while also controlling for random user effects. *N* cases reflects the total number of distinct suicidality/NSSI events in the analysis; *N* observations reflects the total number of observations included in the analysis (i.e., the total number of weeks surrounding suicidality/NSSI that contain data).

**Table S6**

*Estimated Means and Standard Errors (SE) for Post Word Count (Aggregated Weekly) in Proximity to Suicidality and Nonsuicidal Self-Injury (NSSI) Events*

|  | Suicidality (N cases = 159; N observations = 827) | | NSSI (N = cases 43; N observations = 227) | |
| --- | --- | --- | --- | --- |
| Time | Mean | *SE* | Mean | *SE* |
| 3 weeks before | 108.56 | 5.94 | 162.27 | 15.03 |
| 2 weeks before | 100.60 | 5.57 | 60.74 | 7.84 |
| 1 week before | 110.34 | 5.66 | 92.22 | 9.61 |
| 1 week after | 91.34 | 5.36 | 81.74 | 9.17 |
| 2 weeks after | 97.42 | 5.52 | 65.00 | 8.13 |
| 3 weeks after | 100.73 | 5.81 | 58.33 | 7.77 |

*Note.* The means and standard errors presented here have been estimated from the GLMMs, and thus are in accordance with the repeated measures nature of the data (i.e., person-centered) while also controlling for random user effects. *N* cases reflects the total number of distinct suicidality/NSSI events in the analysis; *N* observations reflects the total number of observations included in the analysis (i.e., the total number of weeks surrounding suicidality/NSSI that contain data).

**Figure S3**

*GLMM Descriptive Plots: Changes in Posting Frequency and Post Length in Proximity to Recent Suicidality and Nonsuicidal Self-Injury (NSSI) Events*


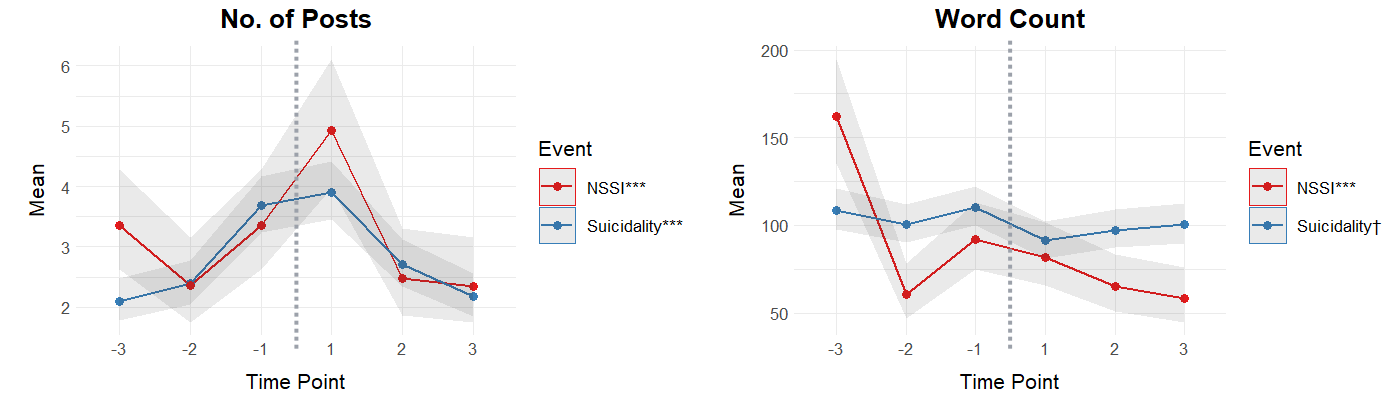


*Note.* The figure shows changes in the mean number of posts made to BPD subreddits (i.e., posting frequency) and the mean word count of posts (i.e., post length) per week (i.e., aggregated weekly) surrounding suicidality and NSSI events. The dotted lines illustrate the point at which engagement in the event occurred (i.e., time point 0), thus dividing the figures by pre- and post-event. The shaded areas surrounding the means represent the error margins (95% confidence intervals). The means (and confidence intervals) have been estimated from the generalised linear mixed models (GLMMs), and thus are reflective of the repeated measures nature of the data (i.e., person-centered) while also controlling for random user effects. The indicators assigned to the suicidality and NSSI keys show the statistical significance of the overall fixed effects of time in proximity to the events: ****p* < .001, ***p* < .01, **p* < .05, ^†^*p* < .10. Time point labels: -3 = three weeks before event, -2 = two weeks before event, -1 = one week before event, 1 = one week after event, 2 = two weeks after event, 3 = three weeks after event.

**Supplemental Materials G:**

**Main GLMM Results Tables and Figures for RQ1**

**Table S7**

*GLMM Descriptive Statistics and Fixed Effects of Time in Proximity to Suicidality (Aggregated Weekly) on Language Variables (N Cases = 159; N Observations = 827)*

|  | Time Point (Estimated Means (SE)) | | | | | |  |  |
| --- | --- | --- | --- | --- | --- | --- | --- | --- |
| LIWC Variable | -3 | -2 | -1 | 1 | 2 | 3 | *F* | *p* |
| I | 9.50 (0.32) | 9.13 (0.30) | 9.50 (0.30) | 9.60 (0.30) | 9.25 (0.30) | 9.70 (0.30) | 0.65 | .662 |
| Negations | 2.52 (0.12) | 2.44 (0.12) | 2.52 (0.11) | 2.51 (0.11) | 2.59 (0.11) | 2.62 (0.12) | 0.34 | .891 |
| Positive emotion | 0.93 (0.08) | 1.06 (0.08) | 0.94 (0.08) | 1.13 (0.08) | 0.92 (0.08) | 0.95 (0.08) | 1.39 | .224 |
| Negative emotion | 1.69 (0.12) | 1.63 (0.11) | 1.92 (0.11) | 1.75 (0.11) | 1.64 (0.11) | 1.77 (0.12) | 1.19 | .315 |
| Anxiety | 0.19 (0.04) | 0.51 (0.05) | 0.41 (0.05) | 0.31 (0.04) | 0.33 (0.04) | 0.48 (0.05) | 7.85 | <.001 |
| Sadness | 0.29 (0.04) | 0.31 (0.04) | 0.50 (0.05) | 0.30 (0.04) | 0.33 (0.04) | 0.28 (0.04) | 6.04 | <.001 |
| Anger | 0.38 (0.05) | 0.30 (0.05) | 0.41 (0.05) | 0.35 (0.05) | 0.37 (0.05) | 0.37 (0.05) | 0.57 | .720 |
| Swear | 0.34 (0.05) | 0.41 (0.05) | 0.51 (0.05) | 0.28 (0.04) | 0.55 (0.05) | 0.36 (0.05) | 7.60 | <.001 |
| We | 0.58 (0.07) | 0.42 (0.06) | 0.48 (0.06) | 0.51 (0.06) | 0.61 (0.06) | 0.39 (0.06) | 2.09 | .065 |
| You | 2.21 (0.18) | 2.28 (0.18) | 2.15 (0.17) | 2.44 (0.18) | 2.28 (0.18) | 2.56 (0.19) | 0.88 | .492 |
| Shehe | 1.41 (0.14) | 1.49 (0.13) | 1.05 (0.12) | 1.10 (0.12) | 1.08 (0.12) | 1.01 (0.13) | 3.27 | 006 |
| They | 0.85 (0.10) | 0.94 (0.09) | 0.95 (0.09) | 0.82 (0.09) | 0.79 (0.09) | 0.90 (0.10) | 0.57 | .725 |
| Affiliation | 1.95 (0.13) | 1.69 (0.13) | 1.70 (0.12) | 2.08 (0.13) | 2.27 (0.13) | 1.78 (0.13) | 4.20 | <.001 |
| Social references | 7.32 (0.31) | 7.82 (0.30) | 7.16 (0.29) | 7.72 (0.29) | 7.34 (0.29) | 7.53 (0.31) | 0.85 | .516 |
| Cognitive processes | 15.90 (0.34) | 16.40 (0.33) | 16.25 (0.32) | 16.37 (0.32) | 16.25 (0.33) | 16.14 (0.34) | 0.35 | .885 |
| Absolutism | 1.68 (0.11) | 1.50 (0.11) | 1.83 (0.11) | 1.75 (0.11) | 1.72 (0.11) | 1.79 (0.11) | 1.29 | .265 |

*Note.* The means and standard errors reported here have been estimated from the generalised linear mixed models (GLMMs), and thus are reflective of the repeated measures nature of the data (i.e., person-centered) while also controlling for random user effects. *N* cases reflect the total number of distinct suicidality events in the analysis; *N* observations reflect the total number of observations included in the analysis (i.e., the total number of weeks surrounding suicidality that contain data). Time point labels: -3 = three weeks before event, -2 = two weeks before event, -1 = one week before event, 1 = one week after event, 2 = two weeks after event, 3 = three weeks after event. *SE* = standard error.

**Table S8**

*GLMM Descriptive Statistics and Fixed Effects of Time in Proximity to Nonsuicidal Self-Injury (Aggregated Weekly) on Language Variables (N Cases = 43; N Observations = 227)*

|  | Time Point (Estimated Means (SE)) | | | | | |  |  |
| --- | --- | --- | --- | --- | --- | --- | --- | --- |
| LIWC Variable | -3 | -2 | -1 | 1 | 2 | 3 | *F* | *p* |
| I | 9.65 (0.58) | 10.35 (0.53) | 9.27 (0.51) | 10.44 (9.25) | 10.17 (0.53) | 9.29 (0.52) | 1.51 | .189 |
| Negations | 2.15 (0.22) | 2.60 (0.20) | 2.73 (0.20) | 2.61 (0.20) | 2.60 (0.20) | 2.70 (0.21) | 0.97 | .436 |
| Positive emotion | 0.99 (0.14) | 0.86 (0.12) | 0.78 (0.12) | 1.12 (0.13) | 1.10 (0.13) | 0.97 (0.13) | 1.28 | .273 |
| Negative emotion | 1.78 (0.25) | 1.67 (0.22) | 1.69 (0.22) | 2.06 (0.22) | 1.61 (0.22) | 1.64 (0.22) | 0.65 | .661 |
| Anxiety | 0.47 (0.09) | 0.39 (0.07) | 0.55 (0.08) | 0.49 (0.07) | 0.21 (0.07) | 0.20 (0.07) | 3.27 | .007 |
| Sadness | 0.31 (0.09) | 0.68 (0.11) | 0.13 (0.05) | 0.14 (0.06) | 0.28 (0.06) | 0.22 (0.06) | 12.74 | <.001 |
| Anger | 0.20 (0.07) | 0.10 (0.06) | 0.09 (0.06) | 0.72 (0.13) | 0.10 (0.06) | 0.19 (0.07) | 12.33 | <.001 |
| Swear | 0.42 (0.08) | 0.29 (0.07) | 0.51 (0.09) | 0.33 (0.07) | 0.34 (0.07) | 0.30 (0.08) | 2.23 | .052 |
| We | 0.30 (0.07) | 0.14 (0.06) | 0.24 (0.09) | 0.79 (0.12) | 0.45 (0.10) | 0.31 (0.07) | 12.58 | <.001 |
| You | 2.35 (0.32) | 1.91 (0.28) | 2.83 (0.32) | 2.07 (0.28) | 2.09 (0.29) | 2.50 (0.30) | 1.95 | .088 |
| Shehe | 0.85 (0.26) | 1.51 (0.26) | 1.62 (0.26) | 0.87 (0.24) | 1.80 (0.27) | 1.30 (0.25) | 2.15 | .061 |
| They | 0.67 (0.11) | 0.54 (0.10) | 0.67 (0.10) | 0.74 (0.11) | 0.50 (0.10) | 0.75 (0.11) | 1.16 | .329 |
| Affiliation | 2.65 (0.26) | 1.62 (0.22) | 1.84 (0.23) | 2.33 (0.23) | 2.18 (0.24) | 2.05 (0.23) | 2.60 | .026 |
| Social references | 7.03 (0.53) | 6.72 (0.49) | 7.93 (0.51) | 7.04 (0.48) | 7.50 (0.51) | 8.28 (0.51) | 1.85 | .104 |
| Cognitive processes | 16.09 (0.61) | 16.08 (0.55) | 16.71 (0.55) | 16.47 (0.55) | 16.75 (0.56) | 16.67 (0.55) | 0.35 | .882 |
| Absolutism | 1.65 (0.17) | 1.43 (0.15) | 1.36 (0.15) | 1.44 (0.15) | 1.42 (0.15) | 1.40 (0.15) | 0.47 | .802 |

*Note.* The means and standard errors reported here have been estimated from the generalised linear mixed models (GLMMs), and thus are reflective of the repeated measures nature of the data (i.e., person-centered) while also controlling for random user effects. *N* cases reflect the total number of distinct NSSI events in the analysis; *N* observations reflect the total number of observations included in the analysis (i.e., the total number of weeks surrounding NSSI that contain data). Time point labels: -3 = three weeks before event, -2 = two weeks before event, -1 = one week before event, 1 = one week after event, 2 = two weeks after event, 3 = three weeks after event. *SE* = standard error.

**Figure S4**

***
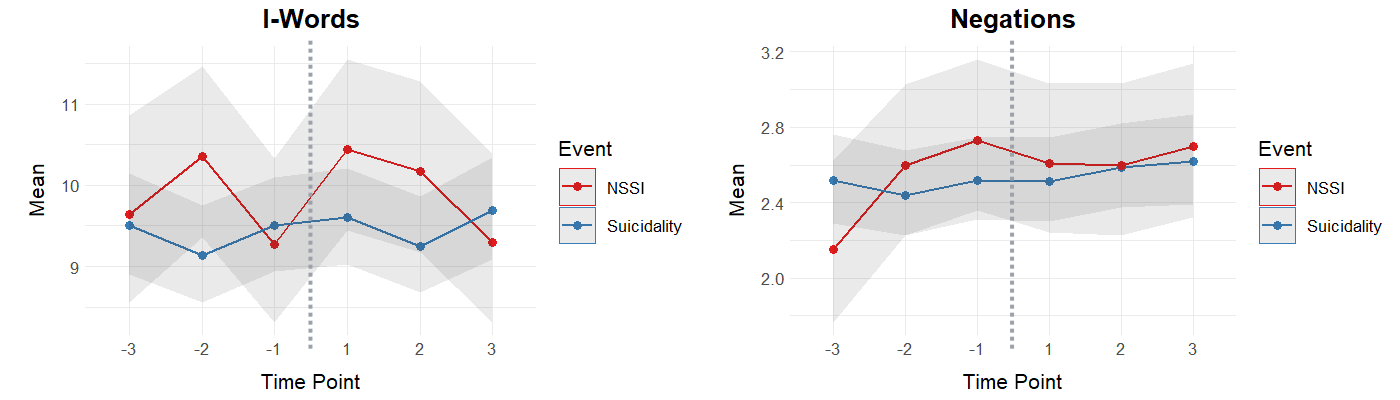
****GLMM Self-Processes Plots: Changes in Mean Self-Processes Language Indicators in Proximity to Recent Suicidality and Nonsuicidal Self-Injury (NSSI) Events*

*Note.* The figure shows changes in mean linguistic indicators of self-processes scores (derived from LIWC) per week (i.e., aggregated weekly) surrounding suicidality and NSSI events. The dotted lines illustrate the point at which engagement in the event occurred (i.e., time point 0), thus dividing the figures by pre- and post-event. The shaded areas surrounding the means represent the error margins (95% confidence intervals). The means (and confidence intervals) have been estimated from the generalised linear mixed models (GLMMs), and thus are reflective of the repeated measures nature of the data (i.e., person-centered) while also controlling for random user effects. There are no significance indicators assigned to suicidality and NSSI keys in this figure as there were no statistically significant overall fixed effects of time in proximity to the events for these variables. Time point labels: -3 = three weeks before event, -2 = two weeks before event, -1 = one week before event, 1 = one week after event, 2 = two weeks after event, 3 = three weeks after event.

**Figure S5**

*GLMM Cognition Plots: Changes in Mean Cognitive Language in Proximity to Recent Suicidality and Nonsuicidal Self-Injury (NSSI) Events*

*
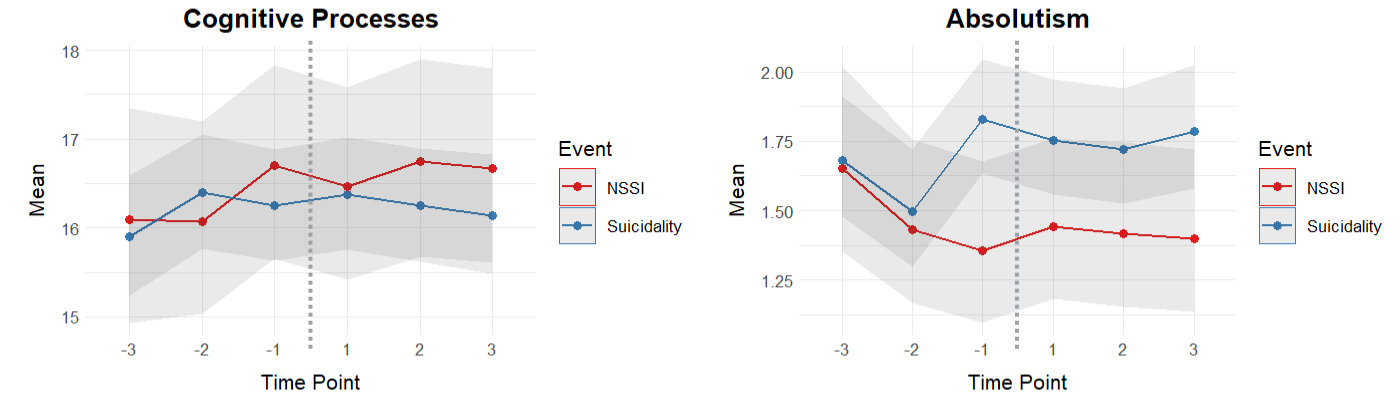
*

*Note.* The figure shows changes in mean cognitive language category scores (derived from LIWC) per week (i.e., aggregated weekly) surrounding suicidality and NSSI events. The dotted lines illustrate the point at which engagement in the event occurred (i.e., time point 0), thus dividing the figures by pre- and post-event. The shaded areas surrounding the means represent the error margins (95% confidence intervals). The means (and confidence intervals) have been estimated from the generalised linear mixed models (GLMMs), and thus are reflective of the repeated measures nature of the data (i.e., person-centered) while also controlling for random user effects. There are no significance indicators assigned to suicidality and NSSI keys in this figure as there were no statistically significant overall fixed effects of time in proximity to the events for these variables. Time point labels: -3 = three weeks before event, -2 = two weeks before event, -1 = one week before event, 1 = one week after event, 2 = two weeks after event, 3 = three weeks after event.

**Supplemental Materials H:**

**RQ3 Correlation Results Table**

**Table S9**

*Spearman’s Rho Correlations Between Key Linguistic Features and Community Support Variables*

| Language Variable | Post Score  (*N* = 66,786) | Number of Replies  (*N* = 7,307) |
| --- | --- | --- |
| Affiliation | .01 | -.01 |
| We | .02*** | -.01 |
| Shehe | .02*** | -.01 |
| Anxiety | .03*** | .02^†^ |
| Sadness | .04*** | .00 |
| Anger | .04*** | .02 |
| Swear | .04*** | -.01 |

****p* < .001, ^†^*p* < .10.

*Note.* All tests are two-tailed. Post scores reflect the number of upvotes a post receives subtracted by the number of downvotes, thereby reflecting the overall “rating” of the post. Number of replies are in relation to submissions only (i.e., not responses to comments), hence the smaller *N.* These analyses were carried out on the full BPD Reddit dataset.
